# ID proteins promote the survival and primed-to-naive transition of human embryonic stem cells through TCF3-mediated transcription

**DOI:** 10.1038/s41419-022-04958-8

**Published:** 2022-06-15

**Authors:** Haibin Jiang, Mingxia Du, Yaning Li, Tengfei Zhou, Jia Lei, Hongqing Liang, Zhen Zhong, Rafia S. Al-Lamki, Ming Jiang, Jun Yang

**Affiliations:** 1 Institute of Basic Medical Sciences, Chinese Academy of Medical Sciences and School of Basic Medicine, Peking Union Medical College, Beijing, China; 2 Department of Physiology and Department of Cardiology of the Second Affiliated Hospital, Zhejiang University School of Medicine, Hangzhou, Zhejiang, China; 3 Department of Pulmonary and Critical Care Medicine, The First Affiliated Hospital of Wenzhou Medical University, Wenzhou, Zhejiang China; 4 Division of Human Reproduction and Developmental Genetics, Women’s Hospital and Institute of Genetics, Zhejiang University School of Medicine, Hangzhou, Zhejiang China; 5 Department of human anatomy and histoembryology, Zhejiang University School of Medicine, Hangzhou, Zhejiang China; 6 Department of Medicine, National Institute of Health Research Cambridge Biomedical Research Centre, University of Cambridge, Cambridge, UK; 7 Department of Gastroenterology of The Children’s Hospital, Institute of Genetics, Zhejiang University School of Medicine, Hangzhou, Zhejiang China

**Keywords:** Phosphoinositol signalling, Embryonic stem cells

## Abstract

Inhibition of DNA binding proteins 1 and 3 (ID1 and ID3) are important downstream targets of BMP signalling that are necessary for embryonic development. However, their specific roles in regulating the pluripotency of human embryonic stem cells (hESCs) remain unclear. Here, we examined the roles of ID1 and ID3 in primed and naive-like hESCs and showed that ID1 and ID3 knockout lines (IDs KO) exhibited decreased survival in both primed and naive-like state. IDs KO lines in the primed state also tended to undergo pluripotent dissolution and ectodermal differentiation. IDs KO impeded the primed-to-naive transition (PNT) of hESCs, and overexpression of ID1 in primed hESCs promoted PNT. Furthermore, single-cell RNA sequencing demonstrated that ID1 and ID3 regulated the survival and pluripotency of hESCs through the AKT signalling pathway. Finally, we showed that TCF3 mediated transcriptional inhibition of MCL1 promotes AKT phosphorylation, which was confirmed by TCF3 knockdown in KO lines. Our study suggests that IDs/TCF3 acts through AKT signalling to promote survival and maintain pluripotency of both primed and naive-like hESCs.

## Introduction

In 1998, researchers established a human embryonic stem cell line in vitro by isolating the ICM of human blastocysts [1]. Human embryonic stem cells (hESCs) differ from mouse embryonic stem cells (mESCs) because they are more similar to epiblasts (primed state), while mESCs are more similar to the inner cell mass (naive state) [2]. The primed and naive states are associated with different gene expression patterns, except for the *OCT4*, *SOX2* and *NANOG* genes. Genes specific to the naive state include *KLF2*, *KLF4*, *KLF17*, *DPPA3*, *DPPA5*, *DNMT3L* and *TFCP2L1*, while genes expressed in the primed state include *FGF5*, *XIST*, *DNMT3A* and *DNMT3B* [3, 4].

Based on the study of the difference between mESCs and hESCs, conventional hESCs (primed state) were induced to transform from the primed state to the naive state [5, 6] and naive hESCs could differentiate into trophoblast stem cells (TSCs) [7]. Furthermore, recent study showed that a structure similar to that of blastocysts could be constructed in vitro based on naive human cells, laying a foundation for recapitulating human early embryonic development in vitro [8, 9]. Thus, exploring the regulatory mechanisms underlying the primed-to-naive cell transformation and the dissolution of pluripotency in hESCs is important for learning early embryonic development and regenerative medicine.

Inhibition of DNA binding proteins (ID, including ID1-ID4) are a family of proteins containing Helix-loop-helix (HLH) domains that regulate gene expression through dominant negative binding with other HLH transcription factors, such as E protein family members (including TCF3, TCF4 and TCF12) to regulate a series of important biological processes [10–13]. In a mESCs culture system, serum BMP4 activate the Smad1/5 signalling pathway, which induced the expression of ID1 to maintain the self-renewal of mESCs by upregulating NANOG [14, 15]. While ID1 and ID3 can be induced by BMP4/Smad1 signalling, they are not the only downstream targets of BMPs [16]. BMP4/Smad1 also cooperate with the LIF/STAT3 signalling pathway to promote the conversion of epiblast stem cells (EpiSCs) to naive pluripotent stem cells (PSCs) [17], but whether ID1 and ID3 play a leading role in these phenomenon is unclear.

In this study, we found that the knockout (KO) of ID1 and ID3 promoted ectodermal differentiation of primed hESCs and prevented the transformation from primed to naive state. Combined with single-cell sequencing, we found that the knockout of ID1/3 downregulated AKT phosphorylation, which promoted pluripotency withdrawal and hindered the transition towards a naive state. Furthermore, we discovered that ID1 and ID3 regulate the expression of MCL1 through the IDs/TCF3 axis and then activate AKT to thereby prevent the dissolution of hESCs pluripotency.

## Results

### ID1 and ID3 are indispensable for the maintenance of hESCs survival and prevention of ectoderm differentiation

**Fig. 1:**
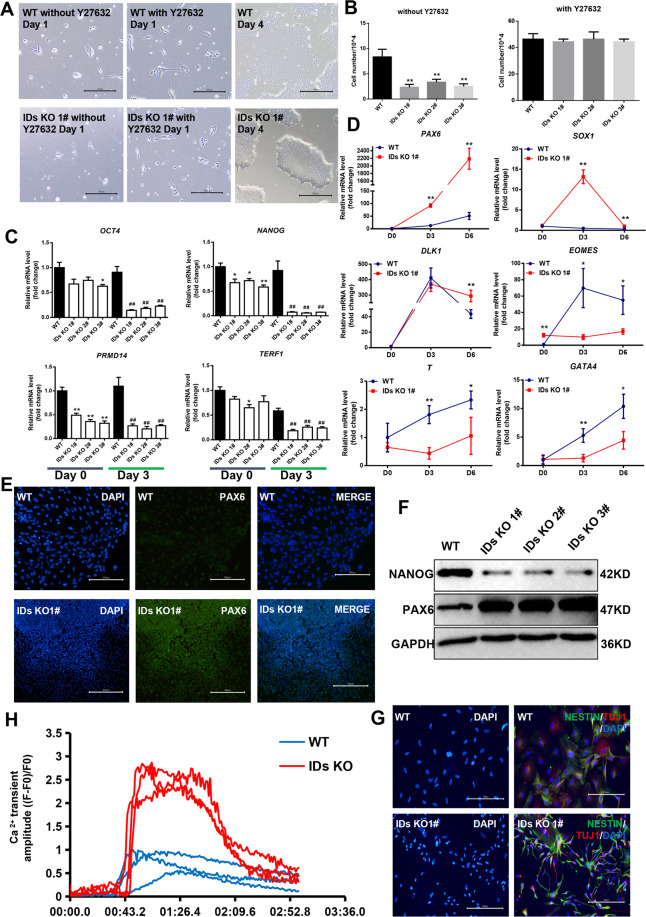
ID1 and ID3 double knockout impairs the survival and promotes ectoderm specification of primed hESCs. **A** Clone morphology of the WT and double knockout lines after culturing for 4 days and the clone outgrowth of the WT and double KO lines after supplementation with or without Y27632, bar = 300 μm. **B** Number of the WT and IDs KO cells after four days of culture with or without Y27632 (*n* = 3), **p < 0.01 compared with WT, *t-*test. **C** Pluripotent markers expression level of the WT and double knockout lines after spontaneous differentiation at day 0 and day 3 (*n* = 3), *p < 0.05; **p < 0.01 compared with day 0 WT; #*p* < 0.05; ##*p* < 0.01 compared with day 3 WT, one-way ANOVA followed by *t*-test. **D** mRNA levels of mesoderm, endoderm, and neuroectoderm markers in the WT and double knockout lines were measured via qPCR at the indicated time points, as the cells spontaneously differentiated into embryoid bodies (EBs); the results were normalized to measurements for the WT cells at the beginning of the differentiation period (*n* = 3). *p < 0.05; **p < 0.01 compared with the WT; one-way ANOVA followed by *t*-test. **E** Immunofluorescence analysis of PAX6 expression levels in the WT and KO lines after spontaneous differentiation, bar = 300 μm. **F** The protien levels of NANOG and PAX6 were detected by immunoblotting after three days spontaneous differentiation. **G** Immunofluorescence analysis of NESTIN and TUJ1 (β3-TUBLIN) expression levels in the WT and double KO lines after spontaneous differentiation, bar = 200 μm. **H** Detection of calcium influx in differentiated neurons after stimulation with glutamic acid.

To clarify the role of ID1 and ID3 in the maintenance of hESCs pluripotency, we examined the expression of IDs in hESCs by immunofluorescence and immunoblotting. High levels of ID1 and ID3 expression were revealed in E8 and mTeSR1 media (Fig. S1A and S1B). To assess the regulatory effects of ID1 and ID3 on hESCs pluripotency, we verified the KO efficiency of three IDs KO lines generated in house from H9 hESCs by immunoblotting (Fig. S1C). Wild type (WT) and IDs KO cells were then dissociated with EDTA and subcultured as single cells. Significantly fewer IDs KO cells than WT cells were present in culture, so we used a Rock inhibitor––Y27632 to enhance cell survival. When cultured with Y27632, the numbers of cells in WT and IDs KO were not significantly different (Fig. 1A, B). These data indicate ID1 and ID3 are indispensable for sustaining the viability of hESCs.

Next, to determine the effect of ID1 and ID3 on the expression of pluripotent genes, real-time PCR (qPCR) was performed and revealed a decrease in the expression of *OCT4*, *NANOG*, *PRMD14* and *TERF1* in IDs KO cells compared with WT cells (Fig. 1C). The levels of pluripotent genes were further reduced on day 3 of differentiation, indicating the double KO of ID1 and ID3 promotes the dissolution of pluripotency.

We next examined the effect of IDs KO on cell fate determination. RNA was collected on Days 0, 3 and 6 of embryoid bodies (EBs) differentiation, and the expression of three germ layer-related genes was detected by qPCR (Fig. 1D and S1D, E). In the early stage of EBs differentiation, the ectoderm-related genes *PAX6*, *DLK1* and *SOX1* were expressed at significantly higher levels in the IDs KO, while the mesoderm-related genes *T* and *EOMES* and the endodermal-related gene *GATA4* were expressed at significantly lower levels in IDs KO. Immunofluorescence staining and western blotting (WB) were performed to detect the protein expression of PAX6 in the early stage of differentiation in the WT and IDs KO lines (Fig. 1E, F), which confirmed that ID1 and ID3 KO promoted the differentiation of ectoderm cells from hESCs. We further performed direct neuronal differentiation and detected the Ca^2+^ transient amplitude after glutamic acid stimulation [18–20]. We found that the KO of ID1 and ID3 promotes the functional neuron differentiation of hESCs (Fig. 1G, H and S2, 3).

### ID1 and ID3 are necessary for resetting hESCs from the primed state to the naive state

**Fig. 2:**
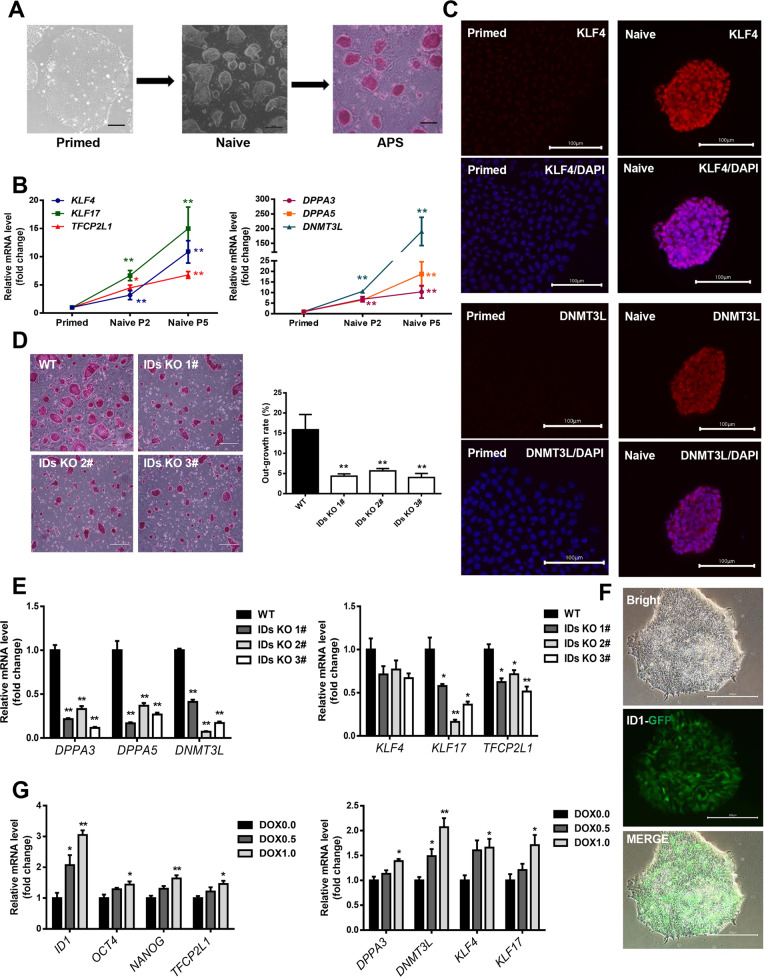
ID1 and ID3 are required for the reset from primed to naive state of hESCs. **A** Reset primed hESCs to a naive state and alkaline phosphatase stain (APS). **B** Naive marker genes expression when primed hESCs reset to naive state (*n* = 3), compared with primed, *p < 0.05; **p < 0.01, data are presented as means ± SEM, one-way ANOVA followed by *t*-test. **C** Immunofluorescence analysis of pluripotency-related gene expression levels in naive hESC, bar = 100 μm. **D** Reset WT and ID1/ID3 KO lines to naive state and the clone out-growth rate of the WT and double konckout lines (*n* = 3), **p < 0.01, data are presented as the means ± SEM, *t*-test. **E** RT-PCR detection of naive gene expression levels in the WT and KO lines (*n* = 3), compared with the WT, *p < 0.05; **p < 0.01, data are presented as means ± SEM, *t*-test. **F** Establishment of the ID1 overexpression line, and the GFP was observed when cultured in E8 with 2 μg/ml DOX, bar = 300 μm. **G** RT-PCR detection of naive gene expression levels after ID1 overexpression line were reset to naive like state and treated with different concentrations of DOX for 48 h (*n* = 3), *p < 0.05; **p < 0.01, compared with DOX 0.0 μg/ml, data are presented as means ± SEM, one-way ANOVA followed by *t*-test.

To determine the role of IDs in naive hESCs, we established hESCs in a naive-like state by using RSeT medium from the Stem Cell^TM^ Technologies (Fig. S4A). As shown in Fig. 2A, the cell morphology was typical of that in the naive state, and alkaline phosphatase staining was positive. In addition, the expression of naive genes in the established cell line such as *DPPA3*, *DPPA5*, *DNMT3L* and *KLF17* was shown to increase significantly as the cells were continuously passaged (Fig. 2B). Next, the expression of pluripotency factors was detected at the protein level (Fig. 2C and S4B, S4C). Then, the WT and IDs KO cells were induced to naive state to observe the effect of ID1 and ID3 KO on the primed-to-naive transition (PNT). After culture in RSeT medium for 2 generations, the clone sizes of the IDs KO cells were significantly smaller and the clone outgrowth rate was markedly lower than those of the WT cells (Fig. 2D). These data indicate that the ID1 and ID3 are essential for promoting the survival ability of naive hESCs. Next, we examined the expression of naive genes in the WT and IDs KO cells by qPCR and found it was significantly lower in IDs KO cells than in WT cells (Fig. 2E), indicating that ID1 and ID3 are indispensable in the PNT of hESCs.

To further understand the role of ID proteins in the PNT, a doxycycline (DOX)-induced ID1-GFP overexpression line was established in our lab (Fig. 2F). Then, the ID1 overexpression line was induced to a naive-like state. As the expression of ID1 increased, the mRNA levels of naive marker genes increased, indicating that ID1 could promote the PNT of hESCs (Fig. 2G).

### Impact of ID1 and ID3 double knockout on the TSCs differentiation potential of naive hESCs

**Fig. 3:**
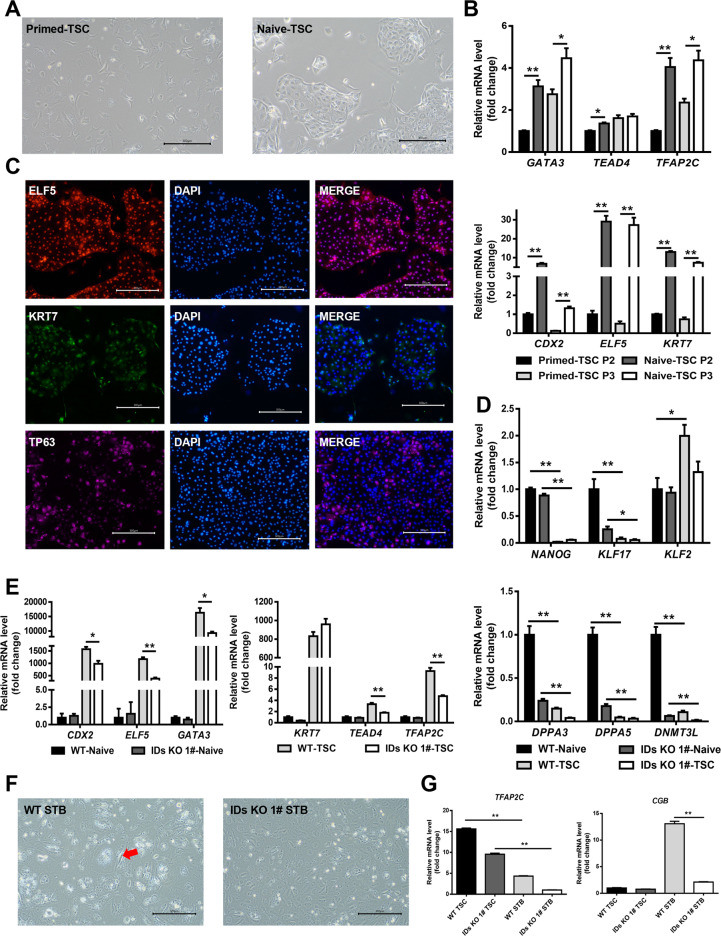
ID1 and ID3 double knockout attenuates the TSC-differentiation potential of naive hESCs. **A** Morphology of trophoblast stem cell that are derived from primed and naive state hESCs separately, bar = 300 μm. **B** Quantitative gene expression analysis for the trophoblast marker genes *ELF5*, *KRT7*, *GATA3*, *TFAP2C*, *TEAD4*,and *CDX2* in the H9 hTSC-like cells derived from naive hESCs, and primed hESCs cultured in hTSC medium (*n* = 3), data are presented as means ± SEM, *t*-test, *p < 0.05; **p < 0.01. **C** Immunofluorescence staining for TSC markers KRT7, ELF5, and TP63 in H9 hTSC-like cells derived from naive hPSCs. The scale bars indicate 300 μm. **D** The naive state associated genes expression level of naive hESCs and TSC that derived from WT and double knockout lines separately (*n* = 3), *p < 0.05; **p < 0.01, compared with WT, data are presented as means ± SEM, one-way ANOVA followed by *t*-test. **E** The TSC associated genes expression level of naive hESCs and TSCs that derived from WT and double knockout lines separately (*n* = 3), *p < 0.05; **p < 0.01, compared with WT, data are presented as means ± SEM, one-way ANOVA followed by *t*-test. **F** Morphology of 2D- syncytiotrophoblast that are derived from WT and double knockout lines TSC separately, bar = 300 μm. **G** qPCR detect the TFAP2C and CGB gene expression level of TSCs and STB that derived from WT and double knockout lines separately, *p < 0.05; **p < 0.01, compared with WT, data are presented as means ± SEM, *t*-test.

To further explore whether the knockout of ID proteins hinders the PNT of hESCs and therefore affects their differentiation potential, we induced the differentiation of WT and IDs KO cells in the naive state into TSCs. First, we established a TSCs differentiation system according to the latest reports [7, 21]. As shown in Fig. 3A, naive hESC-derived TSCs displayed a typical morphology, while primed differentiated TSCs did not. qPCR showed that the expression of TSC marker genes *CDX2*, *KRT7*, *GATA3*, *TFAP2C*, *TEAD4* and *ELF5* was higher in naive-TSC than in the primed-TSCs (Fig. 3B). Then, the TSCs derived from naive hESCs were subjected to immunofluorescence staining to detect the expression of the TSC marker ELF5, KRT7 and TP63 in protein level (Fig. 3C and S4D).

Subsequently, we verified the effect of ID1 and ID3 knockout on the differentiation of naive hESCs into TSCs. Naive WT and IDs KO hESCs were differentiated into TSCs and subcultured to the third generation. qPCR showed the expression levels of the TSC marker genes in the IDs KO cells were significantly lower than in the WT cells, indicating that the knockout of ID1 and ID3 reduced the TSC differentiation potential of naive hESCs (Fig. 3D, E and S4E).

To explore the effect of ID knockout on the differentiation of TSCs, we differentiated TSCs into syncytiotrophoblasts (STBs) (Fig. 3F and G). Morphological assessment of the STBs and qPCR analyses showed that the differentiation of STBs from IDs KO cells was obviously limited. Collectively, these data indicate that the knockout of ID1 and ID3 reduces the differentiation potential of naive hESCs into TSCs and limits the PNT of hESCs.

### ID1 and ID3 double knockout decreases AKT phosphorylation in both primed and naive hESCs

**Fig. 4:**
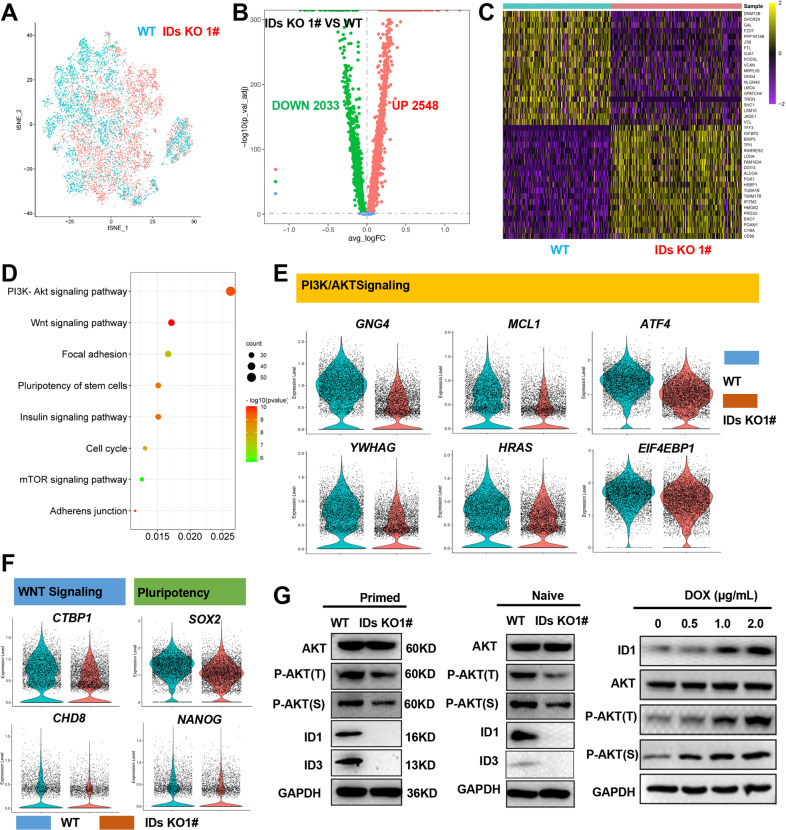
ID1 and ID3 double knockout reduces AKT phosphorylation of hESCs. **A** scRNA-seq t-SNE plot of the WT and double knockout line, WT cells are blue, and double knockout cells are red. **B** Volcano plots showing the fold change (X axis) between the WT and double knockout line cultured in E8 medium. In IDs KO cell, the number of genes with upregulated expression is 2548, and the number of genes with downregulated is 2023. **C** Heatmap shows the distinct gene expression patterns of the WT and IDs KO. Top 20 DEGs are shown. **D** KEGG pathway analysis of the genes with downregulated expression in IDs KO compared with WT. **E**, **F** Violin plots show the expression distributions of specific marker genes that are enriched in PI3K/AKT pathway (**E**), WNT and pluripotency (**F**). **G** Western blotting confirmed the reduction of AKT phosphorylation of both primed and naive state hESCs and the raise of AKT phosphorylation after ID1 overexpression.

To elucidate the underlying molecular mechanism, we conducted single cell RNA sequencing on the WT and IDs KO cells in the primed state, and analysed the results by Seurat. The WT and IDs KO cells were evenly dispersed, and no differentiated cell clusters were found (Fig. 4A). Then, based on differential gene expression between the two lines, a volcano map was constructed (Fig. 4B). A total of 2548 genes had upregulated expression, and 2023 genes had downregulated expression in the IDs KO cells compared to the WT cells. Then top 20 differentially expressed genes (DEGs) were shown in Fig. 4C. These data indicate a change in the gene expression pattern in the IDs KO cells. To illustrate the significance of this change, we performed Kyoto Encyclopedia of Genes and Genomes (KEGG) pathway analysis, which revealed that genes with downregulated expression were enriched in focal adhesion, tight junctions and adherens junctions (Fig. 4D and S5A). Thus, the downregulated expression of genes in these pathways led to a decrease in cell viability (Fig. 1B and S5B). Moreover, some genes with downregulated expression were found to be enriched in the insulin, PI3K/ATK and Wnt signalling pathways. Together, these findings suggest that these pathways are involved in the regulation of hESCs pluripotency. In addition, violin diagrams showed the expression of key genes in the signalling pathways related to regulation of pluripotency (Fig. 4E, F and S6A–C), indicating that IDs may regulated pluripotency through these genes.

Previous reports have shown that insulin/PI3K/AKT signalling pathway inhibit GSK3β to activate Wnt signalling pathway and thereby maintain the pluripotency of hESCs and mESCs [22]. Therefore, we speculated that ID proteins regulate the pluripotency of hESCs through AKT signalling pathway. To assess this hypothesis, we measured phosphorylated AKT in the WT and IDs KO cells by Western blot analysis (Fig. 4G and S6D, S7A, B). The knockout of ID1 and ID3 reduced the level of phosphorylated AKT in both primed and naive hESCs. To further determine the regulatory effect of ID1 and ID3 on AKT phosphorylation in hESCs, ID1 overexpression was induced by DOX, and the results are shown in Fig. 4G, S7C, D. The level of AKT phosphorylation increased as the ID1 expression level increased in primed hESCs. Overall, these results suggest that IDs promote the phosphorylation of AKT in both primed and naive hESCs.

### Inhibition of AKT phosphorylation promotes the ectoderm differentiation of hESCs and impedes their primed-to-naive transition towards TSCs

**Fig. 5:**
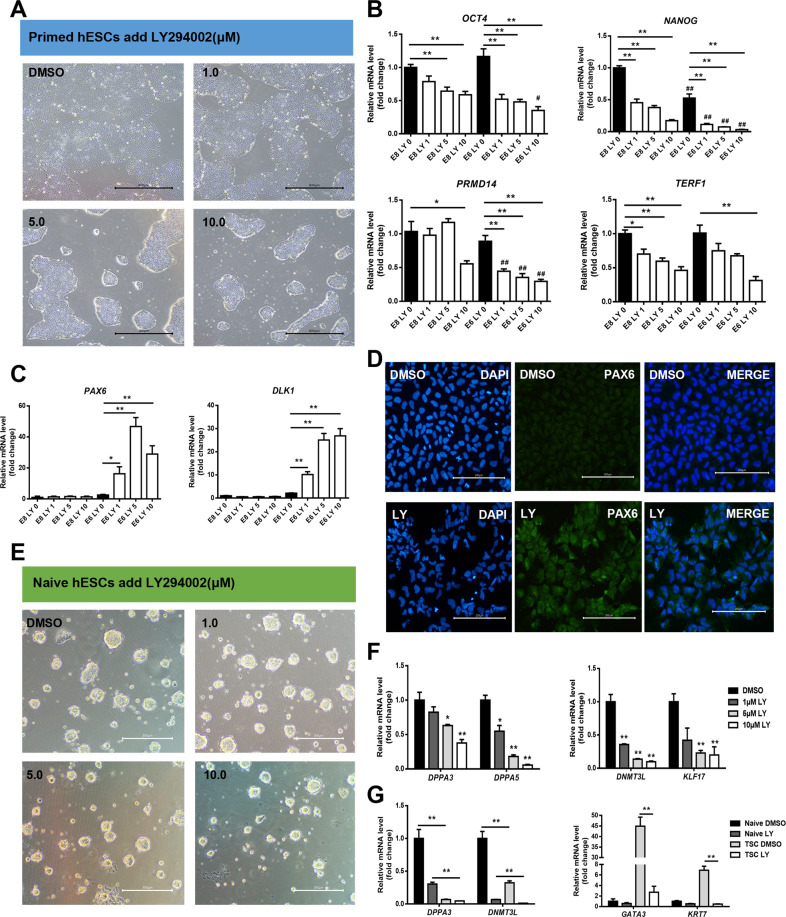
Inhibition of AKT phosphorylation promotes pluripotent dissolution and impedes the primed-to-naive transition. **A** Morphology of primed hESCs after stimulation for 48 h with the PI3K/AKT inhibitor LY294002, at different concentrations bar = 300 μm. **B**, **C** Quantitative gene expression analysis for pluripotent marker genes *OCT4*, *NANOG*, *PRMD14*, and *TERF1* (**B**) and the neuroectoderm marker genes *PAX6* and *DLK1* (**C**) in the primed hESCs after culture for 72 h in E8 and E6 medium with different concentrations of LY294002 separately (*n* = 3), data are presented as means ± SEM, compared to E8 LY 0 and E6 LY 0, *p < 0.05; **p < 0.01, one-way ANOVA followed by *t*-test. **D** Immunofluorescence analysis of PAX6 expression level in primed hESC after cultured 72 h in E6 medium supplemented with 5 μM LY294002, bar = 300 μm. **E** Morphology of naive-like hESCs after stimulation for 48 h with the PI3K/AKT inhibitor LY294002 at different concentrations, bar = 300 μm. **F** qPCR detection of the naive marker genes mRNA levels in naive-like hESCs after treated with different concentration of LY294002 (*n* = 3), data are presented as means ± SEM, compared to DMSO, *p < 0.05; **p < 0.01, one-way ANOVA followed by *t*-test. **G** Naive marker gene and TSC gene expression levels in naive like cells and TSC that treated with LY294002 at different concentration (*n* = 3), data are presented as means ± SEM, compared to DMSO, *p < 0.05; **p < 0.01, one-way ANOVA followed by *t*-test.

Previous studies have reported that phosphorylated AKT inhibited GSK3, subsequently affecting the pluripotency of hESCs and mESCs [22, 23]. However, the regulatory effects of the AKT signalling pathway on termination of pluripotency and the PNT of hESCs are not clear. Therefore, we investigated whether the reduction in AKT phosphorylation by LY294002 parallels that following ID1 and ID3 knockout. First, under E8 and E6 culture conditions, cell morphology was observed after 48 h of culture with LY294002 at different concentrations (Fig. 5A). As the LY294002 concentration increased, the clones gradually shrank. Expression of the pluripotency-related genes in cells cultured under E6 conditions stimulated by LY294002 was decreased to a greater extent than that in cells cultured under the E8 conditions, consistent with the phenotype of ID1 and ID3 knockout, which promoted an exit from the pluripotent state (Fig. 5B).

We next detected the expression of ectodermal genes to confirm that the propensity to differentiate into ectoderm in the IDs KO lines was related to the reduced AKT phosphorylation. The results are shown in Fig. 5C. The addition of LY294002 to the E6 differentiation medium promoted ectoderm differentiation, as confirmed by immunofluorescence staining (Fig. 5D, S7E). These data suggest that differentiation of hESCs to ectoderm caused by an inhibitor-induced reduction in AKT phosphorylation is consistent with that of ID1 and ID3 knockout.

Next, we determined whether the phosphorylation of AKT was altered during the PNT by adding LY294002 at different concentrations to hESCs. Cell morphology analyses demonstrated that the cell clone size decreased as the LY294002 concentration increased (Fig. 5E). Analyses of the expression levels of relevant naive marker genes by qPCR showed that pluripotent marker genes were expressed at a significantly lower level in the cells treated with LY294002 than in control cells (Fig. 5F). Next, we assessed the abilities of naive cells treated with or without LY294002 to differentiate into TSCs, and the results are shown in Fig. 5G. The addition of LY294002 reduced the differentiation potential of TSCs in the naive state. These data indicate that the level of AKT phosphorylation affects the maintenance of hESCs pluripotency and the transition of hESCs to a naive state.

### Identification of MCL1 as a target of the IDs/TCF3 protein axis that regulates AKT phosphorylation

**Fig. 6:**
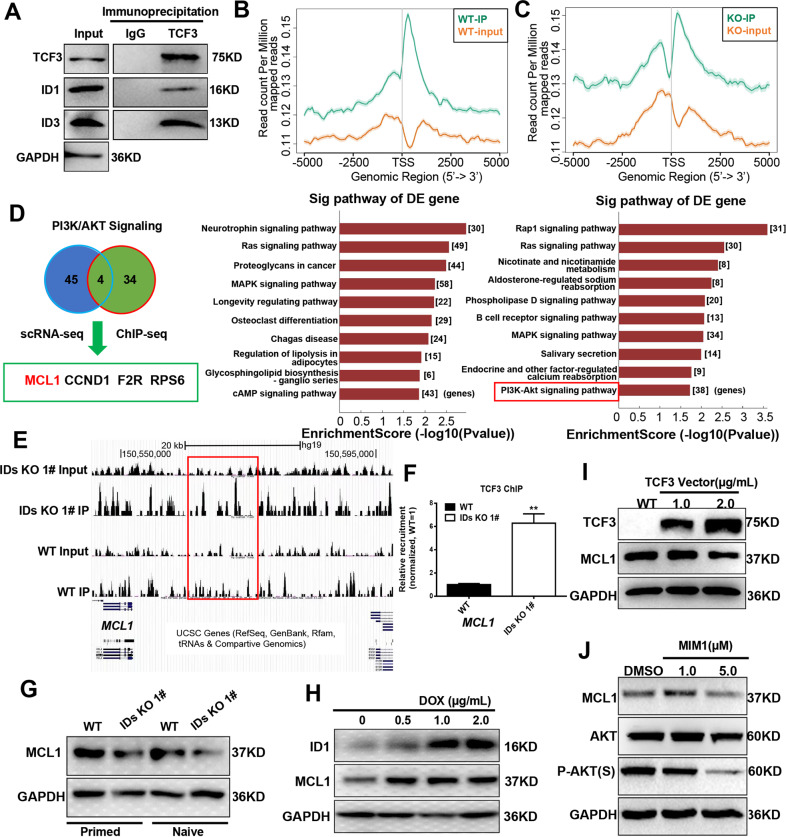
Identify MCL1 as a target of the ID-E protein axis in the regulation of AKT phosphorylation. **A** TCF3 was immunoprecipitated from WT hESCs; then, the amount of ID1 and ID3 present in the precipitate was evaluated via Western blots. **B** Peak annotation plot of TCF3 ChIP-seq analysis in WT and KEGG analysis of the genes that TCF3 binding at in WT. **C** Peak annotation plot of TCF3 ChIP-seq analysis in the IDs KO cells and KEGG analysis of the genes that TCF3 binding at in IDs KO. **D** Screening strategy for identifying potential genes that mediate AKT phosphorylation through TCF3. **E**, **F** TCF3 signal track for the representative locus MCL1 in WT and IDs KO hESCs from ChIP-seq data, and the binding of promoter sequences for MCL1 to TCF3 was evaluated in WT and IDs KO hESCs via ChIP-qPCR (*n* = 3). **p < 0.01 compared with WT; *t*-test. **G** Western blot detect the protein level of MCL1 in both primed and naive-like hESCs. **H** Western blot detect the ID1 and MCL1 expression level in ID1 overexpressed line. **I** Western blot analysis the TCF3 and MCL1 protein level when TCF3 vector was transfect to 293 cell 48 h. **J** Western blot analysis the MCL1, AKT, P-AKT protein level in the cell that treated with different concentrate of MCL1 inhibitor MIM1 48 h.

Next, we investigated the mechanism by which ID1 and ID3 regulate AKT phosphorylation. Previous studies have shown that ID proteins regulate cardiac formation through E protein family members, especially TCF3 (E2A) [24]. In our culture system, we observed the interaction of TCF3 with ID1 and ID3 by Coimmunoprecipitation (Fig. 6A). In addition, we performed TCF3 Chromatin immunoprecipitation (ChIP)-seq to assess differences in the genes pulled down in the WT and IDs KO cells. KEGG analysis of the genes pulled down in the WT and IDs KO cells revealed the associated pathways enriched in these genes (Fig. 6B, C). Previous studies have shown that TCF3 plays a role in transcriptional inhibition and that ID proteins bind TCF3 to inhibit its function [25]. The knockout of ID proteins resulted in the release of TCF3, which binds the transcriptional regulatory region in DNA. Analysis of the results shown in Fig. 6B, C revealed that the PI3K/AKT signalling pathway was enriched in genes expressed by IDs KO cells, and indicated that TCF3 released from ID inhibition, repressed the AKT pathway and thus regulated the expression of related genes. When the genes enriched in the AKT pathway from the single-cell RNA-seq data and ChIP-seq data were put together for comparasion, we identified four overlapping genes (Fig. 6D) that were regulated by TCF3 to subsequently affect the phosphorylation of AKT.

These results, together with previous studies [26], led us to focus on the MCL1 gene. Next, TCF3 signal track and ChIP-qPCR of MCL1 demonstrated that TCF3 binding the upstream of MCL1 locus (Fig. 6E, F). As a recent report has shown that MCL1 can regulate AKT phosphorylation in tumour cells [26], we first confirmed the downregulation of MCL1 expression in the IDs KO lines by WB and qPCR analyses (Fig. 6G and S8A, B). After ID1 overexpression, the analyses revealed that the gene and protein expression of MCL1 increased as that of ID1 increased (Fig. 6H and S8C, D). These data once again demonstrate the regulatory effect of ID1 on MCL1 expression.

Next, we clarified the inhibitory effect of TCF3 on MCL1protein expression. Transfection of the TCF3 plasmid into 293 cells decreased the protein expression of MCL1 (Fig. 6I and S9A, B). Then, we found that the AKT phosphorylation level decreased as the MCL1 expression level reduced in hESCs upon the addition of MIM1 (a specific inhibitor of MCL1 [27]) (Fig. 6J, S9C). These results suggest that MCL1 is a downstream target of IDs/TCF3 and that ID proteins regulate MCL1 and AKT phosphorylation through TCF3.

### Suppressed transcription inhibition of TCF3 on MCL1 increases the pluripotency of IDs KO hESCs

**Fig. 7:**
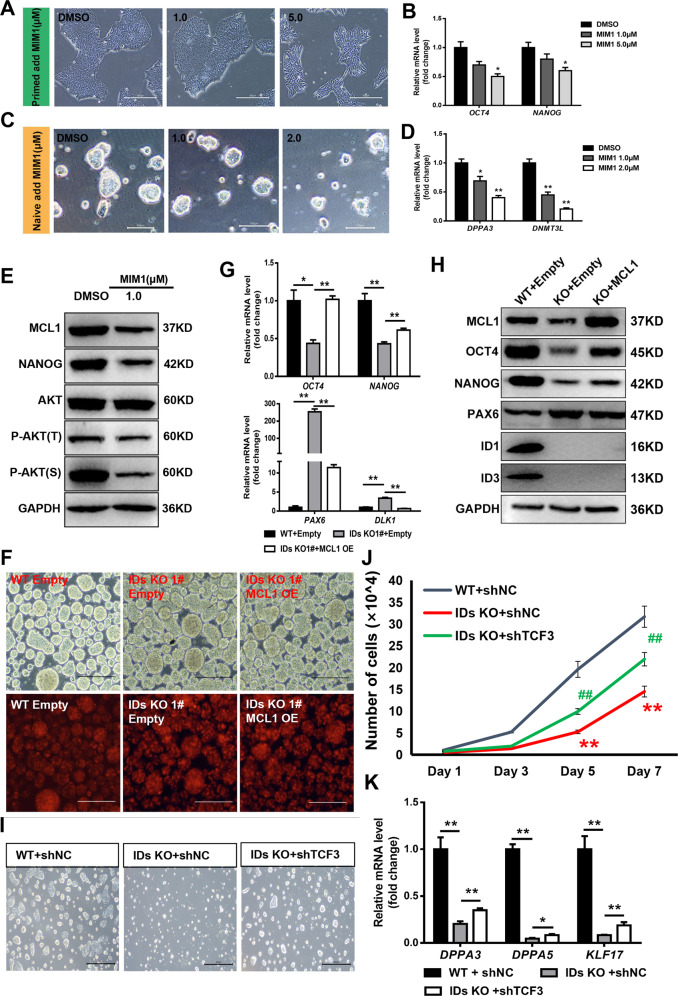
Suppressed transcription inhibition of TCF3 on MCL1 increases the pluripotency of IDs KO hESCs. **A** Morphology of primed hESC after stimulation for 48 h with MCL1 inhibitor MIM1 at different concentrations, bar = 300 μm. **B** Quantitative gene expression analysis of OCT4 and NANOG in the primed hESCs (*n* = 3), compared to DMSO, data are presented as means ± SEM, *p < 0.05, one-way ANOVA followed by *t*-test. **C** Morphology of naive-like hESCs after stimulation for 48 h with the MCL1 inhibitor MIM1 at different concentrations, bar = 300 μm. **D** Quantitative gene expression analysis for DPPA3 and DNMT3L in naïve-like hESC (*n* = 3), compared to DMSO, data are presented as means ± SEM, *p < 0.05, **p < 0.01, one-way ANOVA followed by *t*-test. **E** Western blot analysis of MCL1, NANOG, AKT, and P-AKT protein levels in the naive-like hESCs after treatment with MIM1 for 48 h. **F** Embryonic bodies differentiation of the WT and IDs KO 1# cells at day 3 after transfection with empty and MCL1 overexpression vectors. **G** qPCR detection of pluripotent and ectodermal genes in EBs of the WT and IDs KO 1# cells after transfection with empty and MCL1 overexpression vectors (*n* = 3), compared to WT + empty, data are presented as means ± SEM, *p < 0.05, **p < 0.01, one-way ANOVA followed by *t*-test.. **H** Western blot analysis of MCL1, OCT4, NANOG, and PAX6 protein levels in day 3 EBs of the WT and IDs KO 1# cells after transfection with empty and MCL1 overexpression vectors. **I** Morphology of the WT and IDs KO 1# cells in the naive-like state after separate transfection with shNC (wild type) and shTCF3, bar = 800 μm. **J** Growth curve of the WT and IDs KO 1# cells in naive-like state after separate transfection with shNC and shTCF3, **p < 0.01, compared to WT + shNC, ## *p* < 0.01, compared to IDs KO + shNC, one-way ANOVA followed by *t*-test. **K** Quantitative gene expression analysis in the WT and IDs KO naive-like hESCs after transfection with shNC and shTCF3 (*n* = 3) compared to WT + shNC. Data are presented as means ± SEM, *p < 0.05, **p < 0.01, one-way ANOVA followed by *t*-tests.

To determine whether MCL1 has the same regulatory effect on hESCs, we treated primed and naive hESCs with MIM1. A high dose of MIM1 caused a decrease in the cell boundary and induced differentiation and downregulated expression of pluripotency-related genes in primed cells (Figs. 7A, B). In the naive state, the MCL1 inhibitor decreased the sizes of the cell clones (Fig. 7C). Moreover, *DPPA3* and *DNMT3L*, which are related to naive pluripotency, were significantly downregulated (Fig. 7D). The protein samples were then collected for assessment by WB, which revealed that the MCL1 inhibitor significantly decreased the protein expression of MCL1 and NANOG and decreased the phosphorylation of AKT (Fig. 7E, S10A, B). We aslo conducted MCL1 overexpression in IDs KO lines and found OCT4 and NANOG had partial reversal at mRNA and protein level, meanwhile the expression of *PAX6* and *DLK1* decreased in KO hESCs with MCL1 overexpressed (Fig. 7F–H, S10C). To confirm the transcriptional regulation of TCF3 on MCL1, we knocked down the TCF3 by shRNA in naive-like hESCs and found the expression of MCL1 was increased, which confirmed that the TCF3 was regulated by IDs to repress the transcription of MCL1 (Fig. S10D). Although we couldn’t detect significantly rescued genes expression or other phenotype in IDs KO line, the TCF3 knockdown with shRNA in IDs KO line markedly enhanced the survival and proliferation of IDs KO line compared with shNC treated IDs KO lines at naive-like state (Fig. 7I, J). qPCR showed that the expression of naive marker genes could be partially rescued (Figs. S5D, 7K). We further confirmed that TCF3 inhibited naive gene expression by knocking down TCF3 in hESCs (Fig. S10E). Thus, these results confirm that suppressed transcription inhibition of TCF3 on MCL1 increases the pluripotency of KO hESCs in a naive-like state.

## Discussion

In this study, we found that double KO of ID1 and ID3 significantly reduced the single-cell viability and pluripotency of hESCs. KEGG pathway analysis of the single-cell transcriptome sequencing results showed reduced gene expression in the IDs KO cells compared with the WT cells, and the genes were mainly enriched in the focal adhesion and PI3K/AKT signalling pathways.

During the passage of stem cells, especially at the single-cell level, the stem cell niche is destroyed, then cell adhesion and focal adhesion become defective during this process. At the same time, the Rho-related protein kinase (Rock) signalling pathway is activated, which results in the death of single dissociated cells. The addition of the Rock inhibitor Y27632 to medium promotes the survival of hESCs after passaging [28]. Therefore, after knockout of the ID1 and ID3 proteins, the resultant decreased focal adhesion could not support the hESCs survival-dependent niche, which could be rescued by inhibiting Rock activity.

We investigated the effect of ID knockout on the maintenance of hESCs pluripotency and observed two major events: (a) the differentiation of primed hESCs into the ectodermal lineage and (b) the hampered transition of primed hESCs towards naive hESCs, thus affecting the differentiation of naive hESCs to TSCs. This is the first report that ID1 and ID3 play an essential role in the PNT of hESCs and regulate the TSC differentiation of human embryonic stem cells in the naive state.

In subsequent experiments, we found that ID1 and ID3 knockout significantly reduced AKT phosphorylation in primed and naive cells. Insulin has been shown to maintain the pluripotency of hESCs via activation of the PI3K/AKT patyway in a well-defined medium [29, 30]. LY294002 treatment decreased the expression of pluripotency-related genes in hESCs [31, 32]. The activation of PI3K inhibits the MAPK/ERK signalling pathway, and activated GSK3 leads to the inactivation of β-catenin and potentially inhibits the differentiation of hESCs [23, 33]. Therefore, we identified that ID1 and ID3 regulate viability and pluripotent gene expression in hESCs through the PI3K/AKT signalling pathway.

Using ChIP-seq and scRNA-seq, we identified MCL1 as a target gene regulated by ID proteins that relays the signal needed to activate the PI3K/AKT pathway. MCL1 belongs to the BCL2 protein family, the members of which mainly localize in the mitochondrial membrane [34, 35]. Recent studies have shown that MCL1 directly interacts with AKT, leading to AKT phosphorylation and activation [26]. In hESCs, MCL1 promotes the maintenance of stem cell pluripotency by regulating the mitochondrial dynamics of stem cells [27]. MCL1 knockout mice were shown to have trophectoderm defects that resulted in peri-implantation embryonic lethality [36], consistent with the impaired ability of the IDs KO cells in this study to differentiate into TSCs. In-depth analysis suggested that ID1 and ID3 promote the expression of MCL1 by inhibiting TCF3 and that MCL1 promotes AKT phosphorylation through direct interaction.

**Fig. 8:**
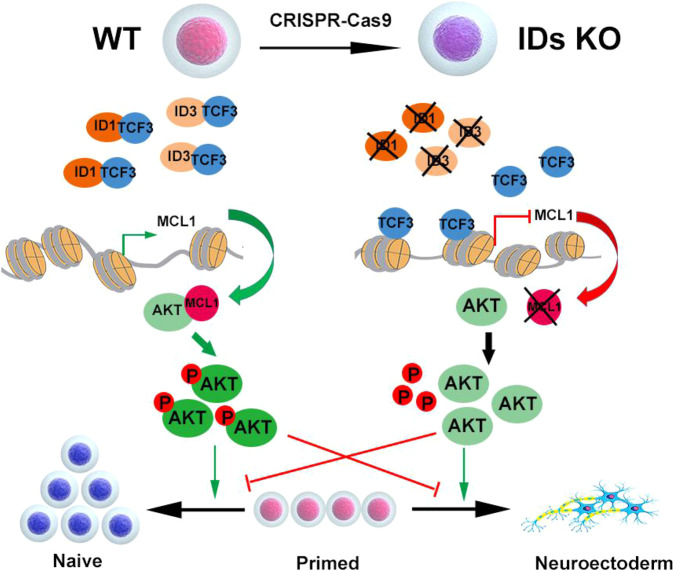
A model of IDs acting through TCF3 to induce AKT phosphorylation to maintain pluripotency in hESCs. Specifically, IDs KO allows TCF3 to bind to the MCL1 promoter region, which represses its expression and thereby reduces the phosphorylation of AKT in hESCs. Consequently, the reduction in AKT phosphorylation promotes the ectodermal differentiation of primed hESCs and inhibits PNT.

In conclusion, the knockout of ID1 and ID3 allows TCF3 to bind the MCL1 promoter region, which represses the expression of MCL1 and thereby reduces the phosphorylation of AKT. The consequent reduction in AKT activity promotes ectoderm differentiation and impairs the transition of hESCs from the primed to the naive state (Fig. 8). Our finding of comparable function of IDs in human naive and primed ESCs confirms that the signalling pathway can reliably be studied in naive hESCs for the application of regenerative medicine.

## Materials and methods

### Cell culture

Human conventional embryonic stem cells (hESCs, H9) were mainly maintained in E8 medium. When the cells were passaged, the original medium was discarded, and the cells were washed with 1 mL of DPBS (12-well plate). Then 500 μL of 0.5 mM EDTA was added, and the cells were incubated at 37 °C for 5 min. Then, the EDTA was discarded and 1 mL of E8 medium was added to suspend the cells. The cells were placed in Matrigel-coated 12-well plates at a ratio of 1:10-1:20. When primed hESCs were reset to a naive state, the cells were resuspended in E8 medium and added to Matrigel-coated 12-well plates. After 24 h, the medium was changed to RSeT medium. After 4 days, the cells were digested with TrypLE for 3 min, and then subcultured at a ratio of 1:10-1:20. The medium was supplemented with 10 μM Y27632. The medium was changed every day, and the cells were passaged the every 3-4 days. All the cell lines we used are mycoplasma clean by PCR detection.

### Chromatin immunoprecipitation assay

The cells were cross-linked with 37% formaldehyde at room temperature for 10 min; then, the reaction was quenched with 0.125 M glycine for 5 min at room temperature. The next experiment was performed as the instructions described (Simple ChIP Enzymatic Chromatin IP Kit, 9003, CST).Briefly, chromatin was digested by micrococcal nuclease for about 20 min at 37 °C to shear chromatin into 150-900 bp fragments. Next, 5-10 μg samples were incubated with 3-5 μg of antibody overnight at 4 °C; 2% of precleared chromatin was reserved for use as input DNA before incubation with the antibody. Next day, protein G was added to the IP samples and incubated for 2 h at 4 °C. At last, the beads were washed, and the chromatin was eluted. For ChIP-qPCR, immunoprecipitated DNA was analysed by qPCR; then, the amplification product was expressed as a percentage of the input and normalized to the control experiment for each condition. DNA libraries from TCF3-ChIP, corresponding to the input DNA samples were prepared and sequenced by Kangcheng BioTechnology Company (Shanghai, China) with an Illumina Genome Analyzer, as directed by the manufacturer’s protocol.

### Statistical analysis

Data are expressed as the mean ± SEM in the Figure legends. Differences between two groups were assayed by one-way ANOVA and two-tailed Student’s *t*-test. The 0.05 level of probability was used as the criterion of significance. Analyses were conducted with GraphPad Prism software. We statistically compared the similar variances between the groups as well. All experiments were repeated at least three times.

The expanded material and methods section is available in the online supplemental material.

## Supplementary information

Authorship confirmation

supplemental information

Reproducibility checklist

Original Data File

## Data Availability

The single cell RNA sequencing data for this paper are publicly available at NCBI Sequence Read Archive under the ID PRJNA761514. The ChIP-seq data for this paper are deposited in GEO database under the accession numbers GSE183518 (reviewer token: qnkjqeakjvibhwd).

## References

[1] Thomson JA, Itskovitz-Eldor J, Shapiro SS, Waknitz MA, Swiergiel JJ, Marshall VS (1998). Embryonic stem cell lines derived from human blastocysts. Science.

[2] Nichols J, Smith A (2009). Naive and primed pluripotent states. Cell Stem Cell.

[3] Takahashi S, Kobayashi S, Hiratani I (2018). Epigenetic differences between naive and primed pluripotent stem cells. Cell Mol Life Sci.

[4] Pfeuty B, Kress C, Pain B (2018). Network features and dynamical landscape of naive and primed pluripotency. Biophysical J.

[5] Takashima Y, Guo G, Loos R, Nichols J, Ficz G, Krueger F (2015). Resetting transcription factor control circuitry toward ground-state pluripotency in human (vol 158, pg 1254, 2014). Cell.

[6] Theunissen TW, Friedli M, He YP, Planet E, O’Neil RC, Markoulaki S (2016). Molecular criteria for defining the naive human pluripotent state. Cell Stem Cell.

[7] Dong C, Beltcheva M, Gontarz P, Zhang B, Popli P, Fischer LA (2020). Derivation of trophoblast stem cells from naive human pluripotent stem cells. Elife.

[8] Yanagida A, Spindlow D, Nichols J, Dattani A, Smith A, Guo G (2021). Naive stem cell blastocyst model captures human embryo lineage segregation. Cell Stem Cell.

[9] Yu LQ, Wei YL, Duan JL, Schmitz DA, Sakurai M, Wang L (2021). Blastocyst-like structures generated from human pluripotent stem cells. Nature.

[10] Desprez PY, Sumida T, Coppe JP (2003). Helix-loop-helix proteins in mammary gland development and breast cancer. J Mammary Gland Biol Neoplasia.

[11] Massari ME, Murre C (2000). Helix-loop-helix proteins: regulators of transcription in eucaryotic organisms. Mol Cell Biol.

[12] Hu WY, Xin YG, Hu J, Sun YX, Zhao YN (2019). Inhibitor of DNA binding in heart development and cardiovascular diseases. Cell Commun Signal.

[13] Roschger C, Cabrele C (2017). The Id-protein family in developmental and cancer-associated pathways. Cell Commun Signal.

[14] Zhao QS, Chang C, Gonzalez JP, Alzahrani K, Button JL, Fraidenraich D (2016). Combined Id1 and Id3 Deletion Leads to Severe Erythropoietic Disturbances. PloS ONE.

[15] Guo ZL, Li HM, Han M, Xu TA, Wu XH, Zhuang YA (2011). Modeling Sjogren’s syndrome with Id3 conditional knockout mice. Immunol Lett.

[16] Morikawa M, Koinuma D, Mizutani A, Kawasaki N, Holmborn K, Sundqvist A (2016). BMP sustains embryonic stem cell self-renewal through distinct functions of different Kruppel-like factors. Stem Cell Rep.

[17] Onishi K, Tonge PD, Nagy A, Zandstra PW (2014). Local BMP-SMAD1 Signaling Increases LIF Receptor-Dependent STAT3 Responsiveness and Primed-to-Naive Mouse Pluripotent Stem Cell Conversion Frequency. Stem Cell Rep.

[18] Grienberger C, Konnerth A (2012). Imaging calcium in neurons. Neuron.

[19] Granzotto A, Canzoniero LMT, Sensi SL (2020). A Neurotoxic Menage-a-trois: Glutamate, Calcium, and Zinc in the Excitotoxic Cascade. Front Mol Neurosci.

[20] Rose CR, Ziemens D, Untiet V, Fahlke C (2018). Molecular and cellular physiology of sodium-dependent glutamate transporters. Brain Res Bull.

[21] Dong C, Fischer LA, Theunissen TW (2019). Recent insights into the naive state of human pluripotency and its applications. Exp Cell Res.

[22] Hishida T, Nakachi Y, Mizuno Y, Katano M, Okazaki Y, Ema M (2015). Functional compensation between Myc and PI3K signaling supports self- renewal of embryonic stem cells. Stem Cells.

[23] Singh AM, Reynolds D, Cliff T, Ohtsuka S, Mattheyses AL, Sun YH (2012). Signaling network crosstalk in human pluripotent cells: a Smad2/3-regulated switch that controls the balance between self-renewal and differentiation. Cell Stem Cell.

[24] Cunningham TJ, Yu MS, McKeithan WL, Spiering S, Carrette F, Huang CT (2017). Id genes are essential for early heart formation. Genes Dev.

[25] Wang LH, Baker NE (2015). E proteins and ID proteins: helix-loop-helix partners in development and disease. Developmental Cell.

[26] Chen G, Park D, Magis AT, Behera M, Ramalingam SS, Owonikoko TK (2019). Mcl-1 interacts with Akt to promote lung cancer progression. Cancer Res.

[27] Rasmussen ML, Kline LA, Park KP, Ortolano NA, Romero-Morales AI, Anthony CC (2018). A non-apoptotic function of MCL-1 in promoting pluripotency and modulating mitochondrial dynamics in stem cells. Stem Cell Rep.

[28] Chan KT, Cortesio CL, Huttentocher A (2007). Integrins in cell migration. Methods Enzymol.

[29] Niwa H, Ogawa K, Shimosato D, Adachi K (2009). A parallel circuit of LIF signalling pathways maintains pluripotency of mouse ES cells. Nature.

[30] Ho L, Tan SYX, Wee S, Wu YX, Tan SJC, Ramakrishna NB (2015). ELABELA is an endogenous growth factor that sustains hESC self-renewal via the PI3K/AKT pathway. Cell Stem Cell.

[31] Kingham E, Welham M (2009). Distinct roles for isoforms of the catalytic subunit of class-IA PI3K in the regulation of behaviour of murine embryonic stem cells. J Cell Sci.

[32] Paling NRD, Wheadon H, Bone HK, Welham MJ (2004). Regulation of embryonic stem cell self-renewal by phosphoinositide 3-kinase-dependent signaling. J Biol Chem.

[33] Davidson KC, Adams AM, Goodson JM, McDonald CE, Potter JC, Berndt JD (2012). Wnt/beta-catenin signaling promotes differentiation, not self-renewal, of human embryonic stem cells and is repressed by Oct4. Proc Natl Acad Sci USA.

[34] Elgendy M, Abdel-Aziz AK, Renne SL, Bornaghi V, Procopio G, Colecchia M (2017). Dual modulation of MCL-1 and mTOR determines the response to sunitinib. J Clin Investig.

[35] Elgendy M, Ciro M, Abdel-Aziz AK, Belmonte G, Dal Zuffo R, Mercurio C (2014). Beclin 1 restrains tumorigenesis through Mcl-1 destabilization in an autophagy-independent reciprocal manner. Nat Commun.

[36] Rinkenberger JL, Horning S, Klocke B, Roth K, Korsmeyer SJ (2000). Mcl-1 deficiency results in peri-implantation embryonic lethality. Genes Dev.

