# Supplementary material for: ID proteins promote the survival and primed-to-naive transition of human embryonic stem cells through TCF3-mediated transcription
Source: Cell Death Dis. 2022 Jun 15;13(6):549. doi: 10.1038/s41419-022-04958-8 (PMC9198052; doi:10.1038/s41419-022-04958-8)

Confirmation email from all authors about the final author list.

Jun Yang#


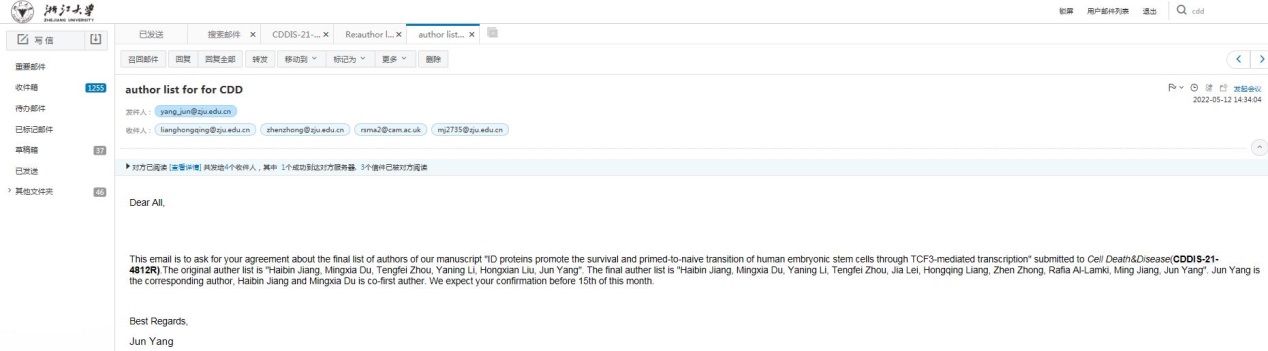


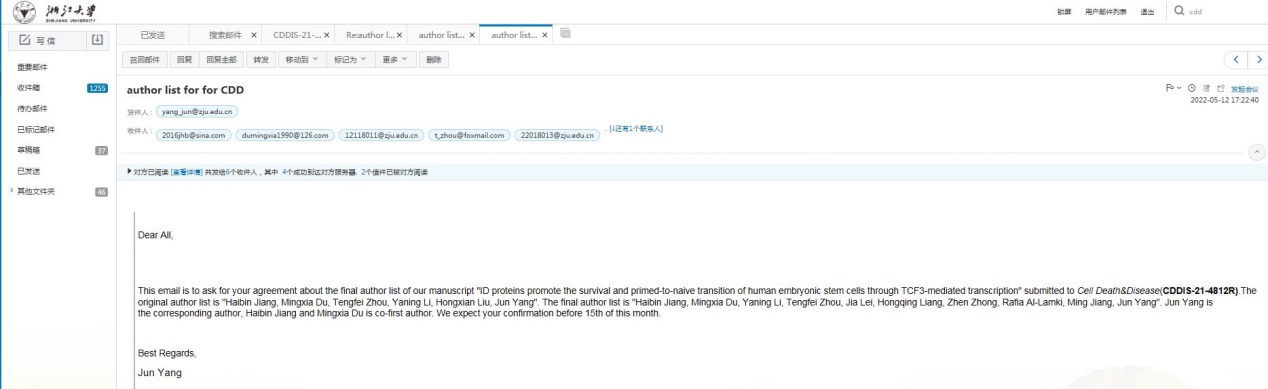


Haibin Jiang


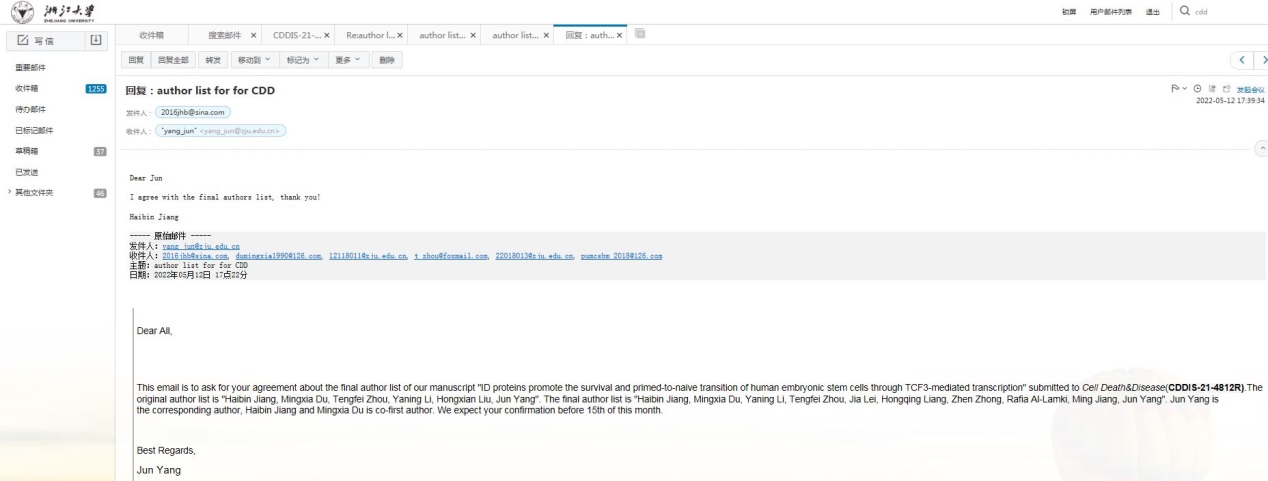


Mingxia Du


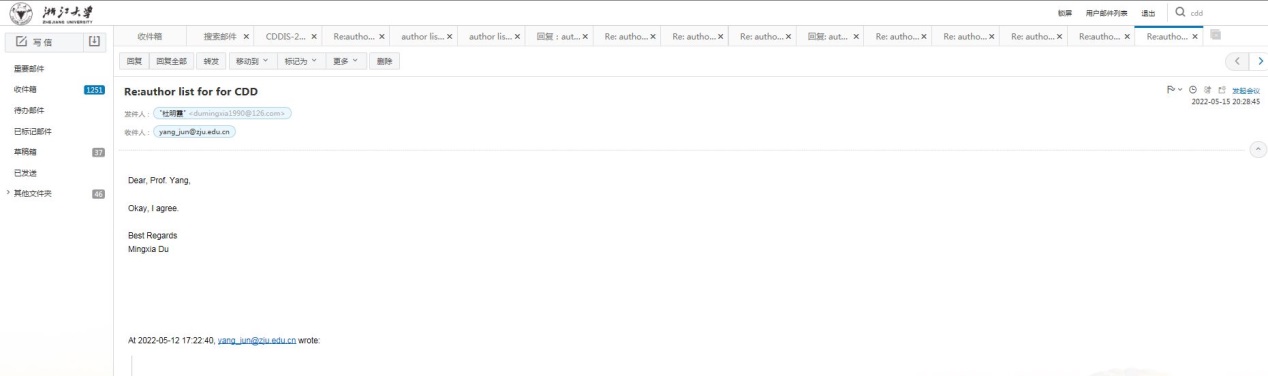


Yaning Li


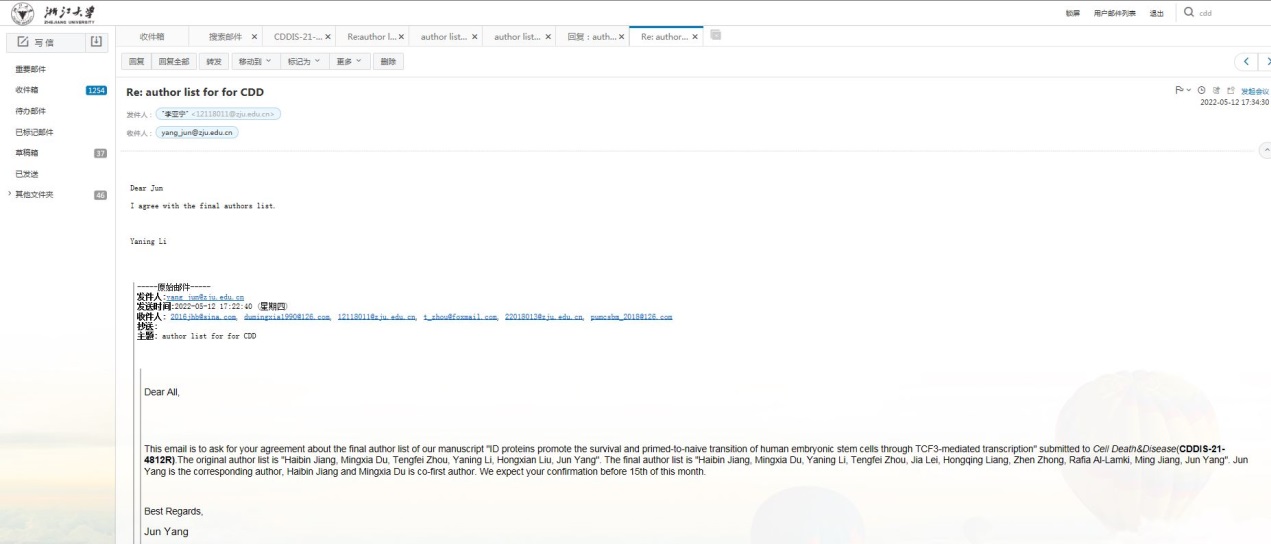


Tengfei Zhou


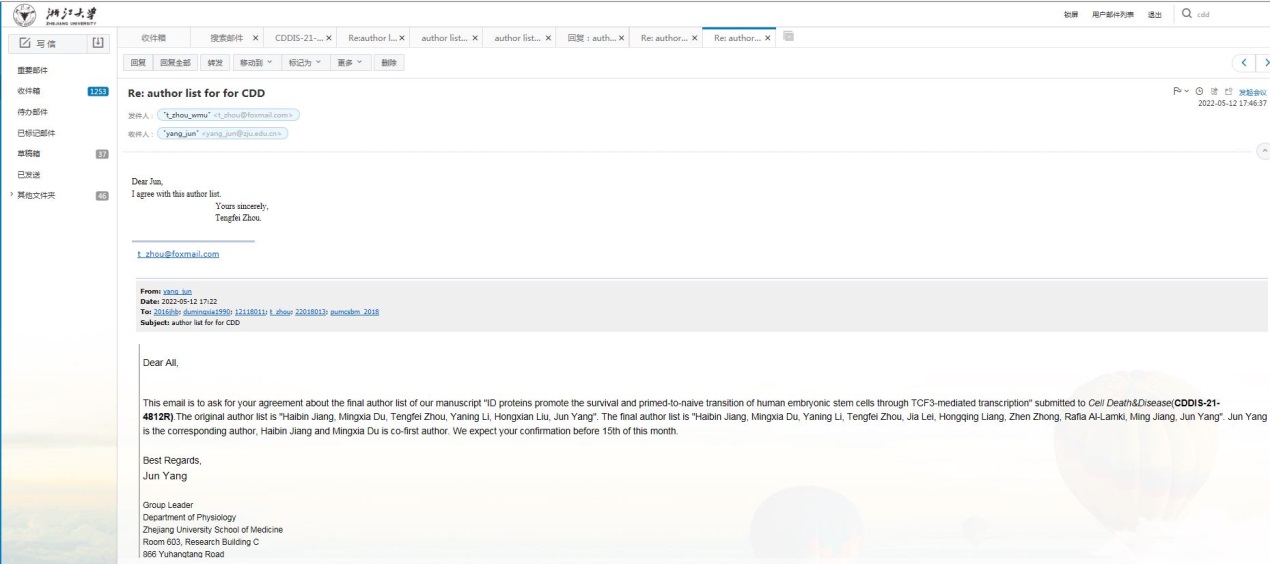


Jia Lei


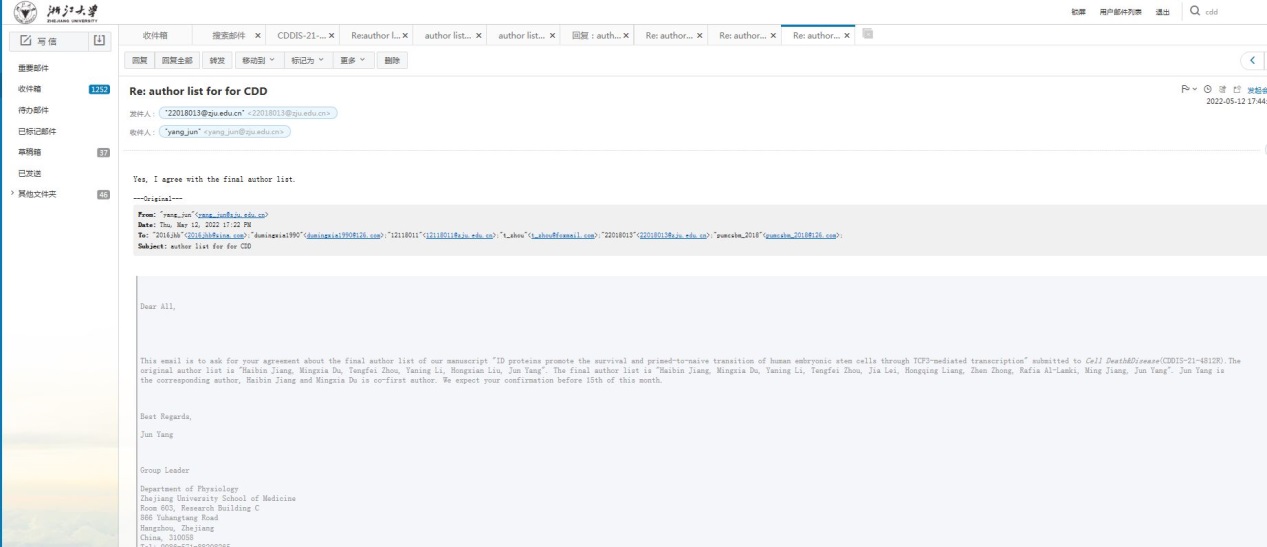


Hongqing Liang


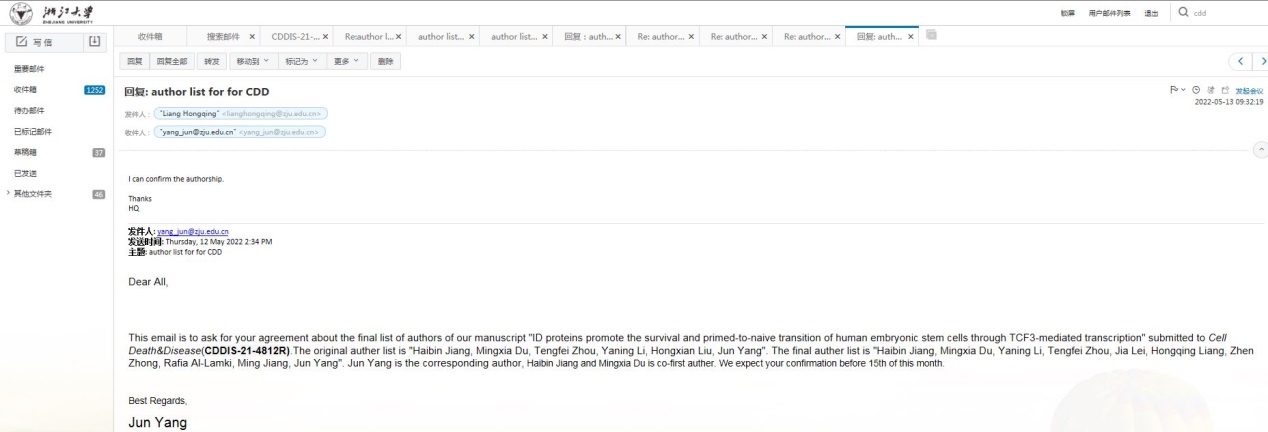


Zhen Zhong


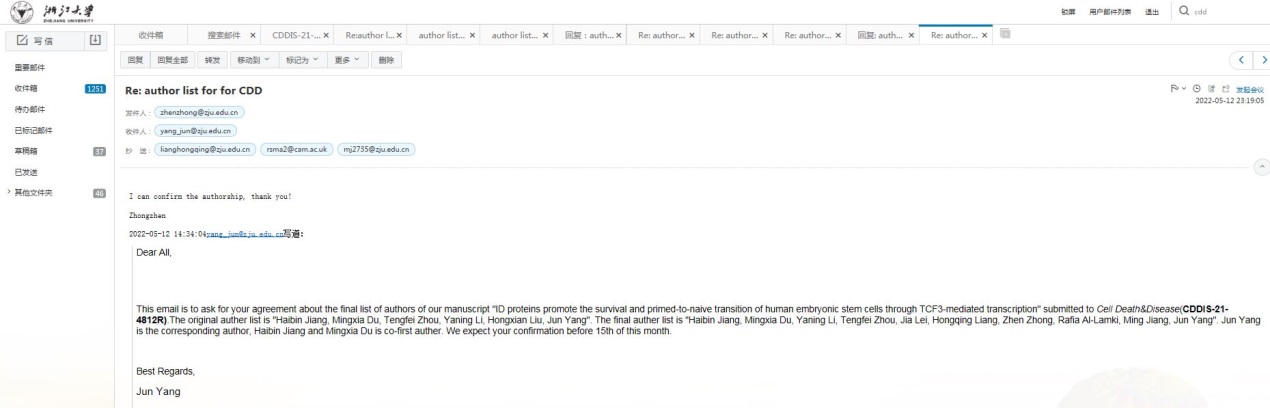


Rafia Al-Lamki


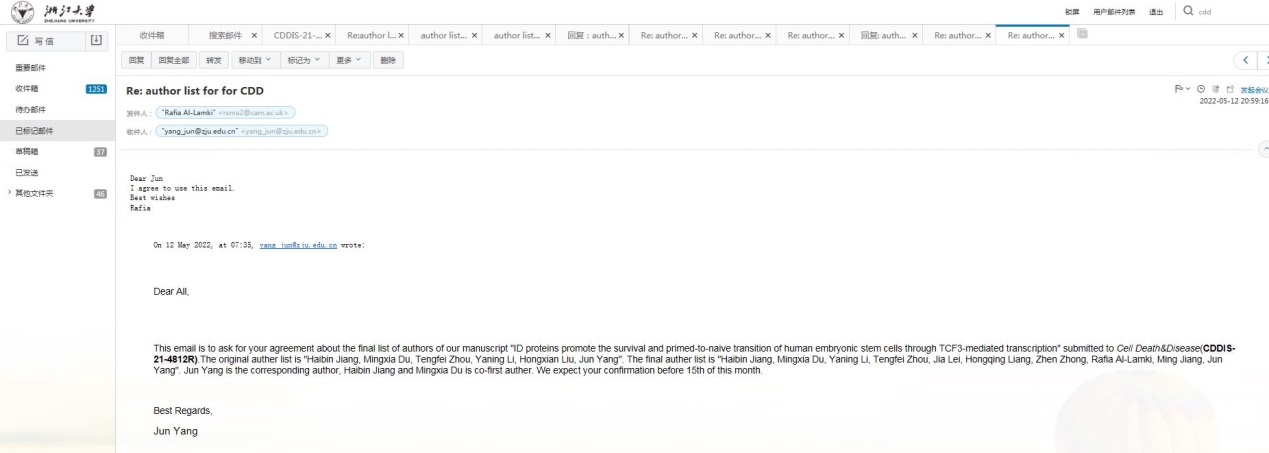


Ming Jiang


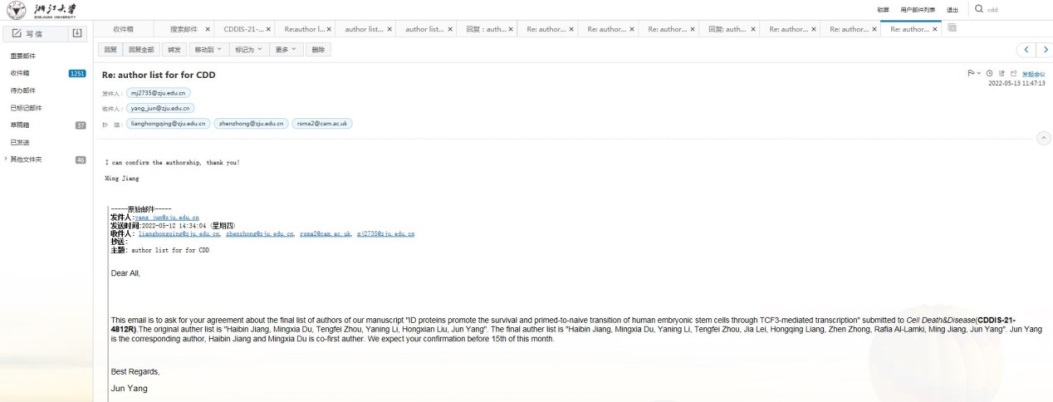


Hongxian Liu


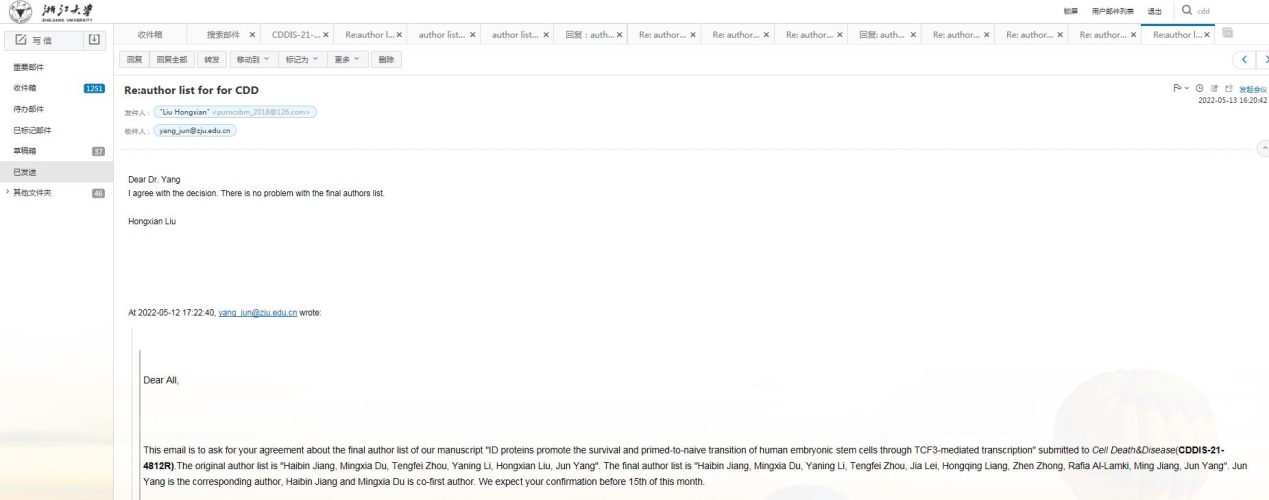

Supplement: Supplementary file 1 — Authorship confirmation [file 41419_2022_4958_MOESM1_ESM.docx]
