# Supplementary material for: ID proteins promote the survival and primed-to-naive transition of human embryonic stem cells through TCF3-mediated transcription
Source: Cell Death Dis. 2022 Jun 15;13(6):549. doi: 10.1038/s41419-022-04958-8 (PMC9198052; doi:10.1038/s41419-022-04958-8)
Supplement: Supplementary file 2 — supplemental information [file 41419_2022_4958_MOESM2_ESM.pdf]

Supplemental Information

Supplemental Material and Methods

Reagents

Essential 8 (Gibco, A1517001), Essential 6 (Gibco, A1516401), DMEM/F12 (Gibco, 11330-032), mTeSR1 (Stemcell Technologies, 85851), RSeT (Stemcell Technologies, 05975), 2-Mercaptoethanol (Gibco, 21985023) , TrypLE Express (Gibco, 12605-010), EDTA (Thermo Scientific, R1021), Matrigel (Corning, 354277), Opti-MEM (Gibco, 11058021), DMSO (Sigma, D2650), LY294002 (Sigma, L9908), MIM1 (MCE, HY-16695), SB431542 (Selleck, S1067), CHIR99021 (Stemcell Technologies, 72052), A83-01 (MCE, HY-10432), Valproic acid (MCE, HY-10585), Forskolin (MCE, HY-15371), Y27632 dihydrochloride (MCE, HY-10583), Recombinant Human EGF (Solarbio, P00033), Doxycycline (Sigma, D9891), Mitomycin C (R&D, 3258/10), Pierce BCA Protein Assay Kit (Thermo Scientific, 23227), HiScript III 1st Strand cDNA Synthesis Kit (Vazyme, R312-02), SYBR qPCR Master Mix (Vazyme, Q311-02), Paraformaldehyde (PFA; Sigma, P6148), Triton X-100 (aMReSCO, 0694), DPAI (Sigma, D9542), PureYield™ Plasmid Miniprep System (Promega, A1223).

Genetic edition of hESCs

The IDs KO and ID1 over-expression lines were previously established in our laboratory. Briefly, IDs KO hESCs were generated with CRISPR-Cas9 genome

editing technology. Single guide RNAs (sgRNAs) targeting the genomic regions of interest were designed with the CRISPR Design Tool (https://zlab.bio/guide-design-resources) and cloned into epiCRISPR vector and transfected into hESCs via electroporation, then selected with puromycin. Positive clones were identified by sequencing the gene of interest and confirmed by analysing protein levels. DOX-inducible ID1 over-expression hESCs were generated with the lentivirus-based system. ID1 complementary DNA (cDNA) was cloned into the vector (pLVX-TRE3G-3 × FLAG-GFP) and transfected into hESCs; then, transfected cells were isolated with neomycin selection, and clones were chosen for use in subsequent experiments.

Alkaline phosphatase staining (APS) of hESCs

hESCs were cultured on Matrigel-coated 12-well plates for 3 days, and AP staining was performed with the Alkaline Phosphatase Kit (Millipore, SCR004). Briefly, the cells were fixed with 4% paraformaldehyde for 2 min at room temperature and washed three times with 1×Rinse Buffer (TBST: 20 mM Tris-HCl, pH 7.4, 0.15 M NaCl, 0.05% Tween-20). Then, 500 μL of Fast Red Violetsolution, 250 μL of Naphthol solution and 250 μL of H_2O were mixed at room temperature and added to the wells. The cells were incubated in the dark at room temperature for 15 min, then rinsed with 1×Rinse Buffer, covered with PBS, and observed under a microscope.

In vitro differentiation of hESCs

For in vitro spontaneous differentiation, hESCs were dissociated with 0.5 mM EDTA at 37°C for 5 min and resuspended in E8 medium at a density of 1.0×10^5cells/mL. Then, the cells were cultured on Ultra Low Cluster Plates in E8 with 10 μM of the Rock inhibitor Y27632 supplemented for 1 day and the EBs were suspended and precipitated in a 15 mL centrifuge tube for 2 min. The supernatant was discarded and the EBs were suspended in E6 medium supplemented with 20% foetal bovine serum, and cultured for 2 days. EBs were transferred to new cell culture plates and cultured until the indicated time points.

Neuron differentiation and Ca^{2+} detection

The differentiation of hESCs into neurons was induced via a NeuroEasy Human Neural Stem Cell Induction Culture Kit (Cellapy, CA2306100), NeuroEasy Human Neural Stem Cell (adherent) Culture Kit (Cellapy, 23014100) and NeuroEasy Human Neuron Differentiation Kit (Cellapy, 2307100). In brief, hESCs were dissociated by 0.5 mM EDTA and seeded on 12-well plates precoated with Matrigel at a ratio of 1:15 in E8 medium. Approximately 3–4 days later, the E8 medium was changed to neural stem cell (NSC) induction medium when the cells reached > 95% confluence. Approximately 3 days later, fresh medium was added to the cells for further culture. When a rosette appeared, the induction of NSCs was complete. Then, the NSCs were passaged for further culture. After 2–3 passages, the NSCs were used for

neuron differentiation. After NSCs were dissociated into single cells, they were moved to a precoated plate, and the medium was changed to neuron induction medium. After 8 days of continuous cultivation, the neurons could be analysed. Differentiated hESC-NSCs were seeded on a glass-bottom cell culture dish with neuron induction medium. After 9–10 days of culture, neuronal cells appeared and matured. Then, 5 μM Fluo-4 AM (Beyotime, S1060) in DPBS was added for 45 min at 37°C. The cells were washed 3 times with DPBS and then incubated for more than 10 min in DPBS to ensure that Fluo-4 AM was completely converted into Fluo-4. Then, a final concentration of 1.5 mM $CaCl_2$ was added to the DPBS before imaging. An inverted confocal microscope (Nikon, Nikon A1R) was used to observe calcium signalling in hESC neurons using a 488 nm single photon laser. The scanning object was determined under the objective lens with 20 times the long working distance and scanned for 30 s to obtain the resting data defined as F0. Then, glutamic acid was added to the dish at a final concentration of 1 mM and scanned for 2 min constantly at a speed of 1–2 images/s. Usually, 100–200 images were collected continuously without a time interval for one region of interest (ROI). Ca^{2+} variation was normalized to the average resting fluorescence intensity (F0) to first obtain F-F0. The Ca^{2+} transient properties were evaluated from (F-F0)/F0.

TSC and STB differentiation of naive hESCs

For TSC differentiation, naive hESCs were dissociated by TrypLE Express for

3-5 min, and 5.0×10^5 cells were seeded on a 12-well plate pre-coated precoated with 1% Martrigel for at least 1 h and cultured in 1 mL of TSC medium. The medium was changed every 2 days, and the cells were passaged upon 80–100% confluency at a ratio of 1:2 to 1:4. After 2 generations of culture, the cells were ready for downstream analysis. TSC medium was as follows: DMEM/F12 supplemented with 0.1 mM 2-mercaptoethanol, 0.2% FBS, 0.5% Penicillin Streptomycin, 0.3% BSA, 1% ITS-X, 1.5 mg/mL L-ascorbic acid, 50 ng/mLEGF, 2 mM CHIR99021, 0.5 mM A83-01, 1 mM SB431542, 0.8 mM VPA (Tocris, 2815), and 5 mM Y-27632.

For 2D STB differentiation, 12-well plates were coated with 500 μL of 2.5 mg/mLCollagen IV overnight. Then, 0.5×10^5 hTSCs were seeded per well in 1 mL of 2D STB medium (DMEM/F12 supplemented with 0.1 mM 2-mercaptoethanol, 0.5% penicillin-streptomycin, 0.3% BSA, 1% ITS-X, 2.5 mM Y-27632, 2 mM Forskolin, and 4% KSR). The mediumm was changed at day 3, and at day 6 the cells were ready for nest detection.

Quantitative real-time PCR assays

Total RNA was extracted using TRIzol reagent, and cDNA was generated by using the HiScript III 1st Strand cDNA Synthesis Kit (Vazyme, R312-02). Real-time qPCR was performed with SYBR qPCR Master Mix (Vazyme, Q311-02), and the primer sequences are listed in Table S1. The mixture was heated to 95°C for 2 min and cycled 40 times (95°C for 30 s, 58°C for 20 s, and 72°C for

10 s). Melting curves were generated by increasing the temperature from 65°C to 95°C in 0.5°C increments at 5-s intervals to ensure that a single peak was visually present for each primer. Threshold amplification values (Ct) were assigned by CFX Manager analysis software (Bio-Rad) and the figures were generated by graphpad.

Immunoblotting and immunoprecipitation (IP)

For protein extraction, the adherent cells were washed with DPBS, and 200 μL of lysis buffer (0.5% Nonidet P- 40, 50 mM Tris, pH 7.6, 120 mM NaCl, 1 mM EDTA, and 1 mM-2-mercaptoethanol) supplemented with 0.1 mM phenylmethylsulfonyl fluoride, 1 mM protease inhibitor cocktail and 1 mM phosphatase inhibitor cocktail was added. Cells were incubated on ice for 10 min and centrifuged for 20 min at 14000×g, at 4°C. Then, the supernatant was separated for subsequent detection. For IP, 1 mg of total protein was incubated with 1 to 10 μg of antibody at 4°C overnight and then, Protein G was added at 4°C for an additional 4 h. Finally the precipitates were washed three times with DPBS with 0.02% Tween 20 and resuspended in 40 μL of 1× SDS loading buffer, and immunoblotting was performed via standard protocols. The following antibodies that used for western blotting are listed in supplementary Table 2.

Immunofluorescence

The cells were seeded on Matrigel-coated microscope cover glass in 24-well

plates, the supernatant was discarded and the cells were fixed with 4% paraformaldehyde for 10 min at room temperature, washed three times for 5 min with DPBS, blocked with 0.2% Triton X-100 and 5% donkey serum in PBS at room temperature for 1 h, then incubated overnight after supplemented with primary antibodies at 4°C, washed with PBS, incubated with secondary antibodies for 1 h at room temperature in the dark, washed with PBS, and then stained with DAPI for 15 min at room temperature. Images were captured with an Olympus microscope. The following antibodies that used for immunofluorescence staining are listed in supplementary Table 2.

Analysis of single cell RNA-seq data

We processed WT and IDs KO samples for single cell capture and cDNA library construction with a 10 × Genomics system (technical support was provided by Novogene Company). The prepared libraries were then sequenced on the Illumina NovaSeq 6000 platform. With the STAR (v2.5.1) tool, the resulting raw data was mapped to the human genome reference GRCh38. In addition, Seurat (4.0.3) was employed to analyse the scRNA-seq data. For quality control, we filtered the cells for the following parameters: mitochondrial genes ≤ 20% and feature counts ranging from 200 to 2500. After normalization for the expression data of the filtered cells, PCA reduction and tSNE clustering (resolution = 0.6) was conducted. In addition, we identified differential expressed genes (DEG) with Log2 (fold change) > 1 and -Log 10 (adjusted P-value) > 0 by using DEseq2

tool. KOBAS (http://kobas.cbi.pku.edu.cn) was used to perform KEGG pathway analysis with the DEGs. T-SNE plots and violin plots and were generated using the TSNEPlot and VlnPlot functions in Seurat. Volcano plots and bubble plot for the visualization of the DEGs and the significant KEGG pathways (ranked by gene ratio, count and adjusted p-value) were generated using the R package ggplot2. Furthermore, a heatmap was drawn to generally show the expression of significant DEGs contributing to the top pathways in the WT and IDs KO.

Supplemental Figures and Legends

Supplemental Figure 1

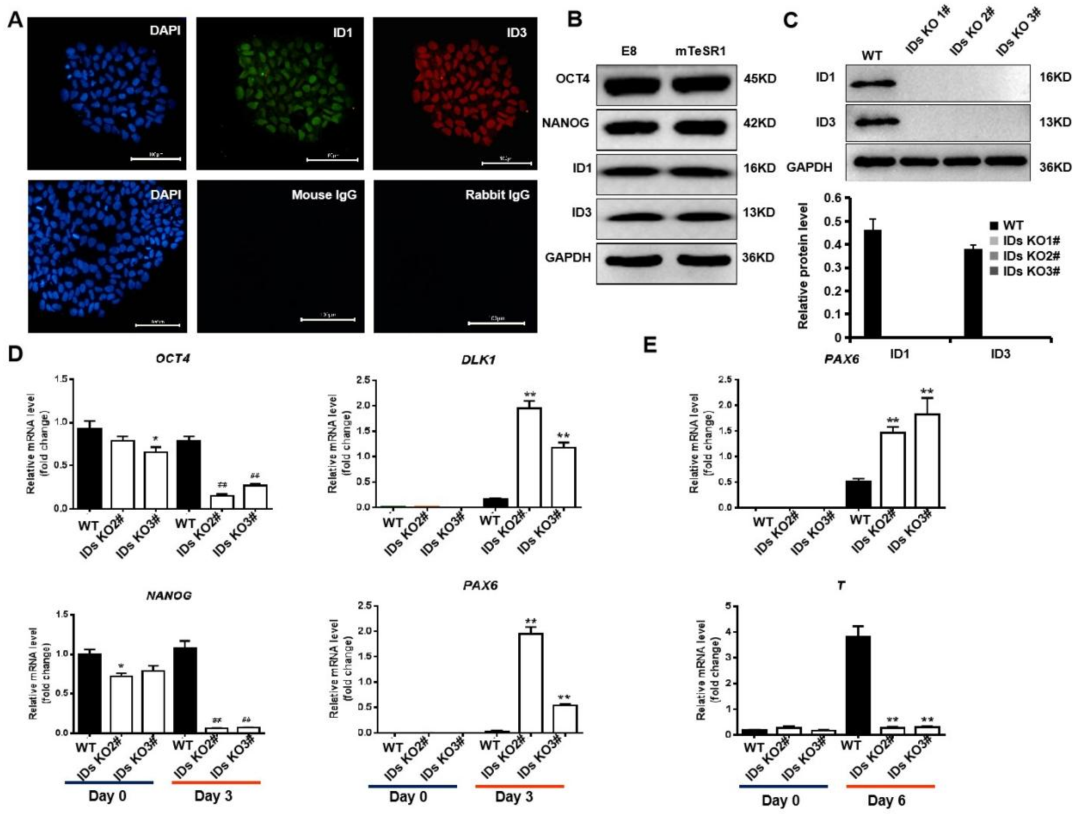

Fig. S1. Knockout of ID1/ID3 leads to spontaneous differentiation of hESCs.

A Immunofluorescence analysis of ID1 and ID3 expression in hESC lines. **B** Immunoblotting analysis of ID1 and ID3 protein level in hESCs cultured at E8 and mTeSR1 conditions. **C** The protein expression levels of ID1 and ID3 were detected by immunoblotting followed with quantitative analyses in ID1 and ID3 double KO lines IDs KO 1#, IDs KO 2#, IDs KO 3# generated in house. **D E** Genes expression level of WT and IDs KO lines after spontaneously differentiated at day 0, day 3, and day6 (N=3), *p<0.05; **p<0.01 compared with day0 WT; #p<0.05; ##p<0.01 compared with day3 or day6 WT, data are presented as means ± SEM, one-way ANOVA followed by t-test.

Supplemental Figure 2

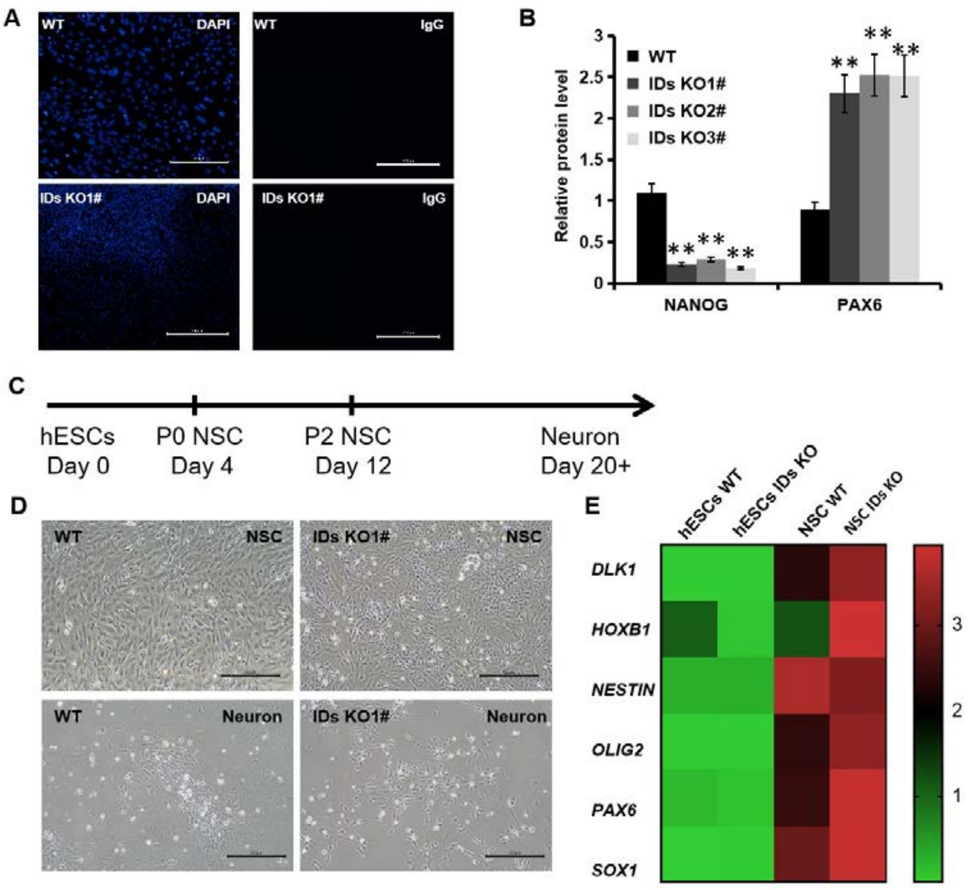

Fig. S2. Neural differentiation of WT and ID1&3 KO hESCs in vitro.

A Immunofluorescence of isotype IgG in WT and ID1&3 KO after differentiation. **B** Quantitative analyses of western blotting (normalized to GAPDH) in figure 1 F (N=3), **p<0.01 compared with WT, data are presented as means ± SEM, one-way ANOVA followed by t-test. **C** Flow diagram of differentiation from hESCs to neuron. **D** Morphology

of neuron stem cell (NSC) and neuron derivated from WT and IDs KO hESCs, bar=300μm. **E** Heat map demonstration of RT-qPCR results by comparing the differential expression of neuron stem cell marker genes between WT and IDs KO.

Supplemental Figure 3

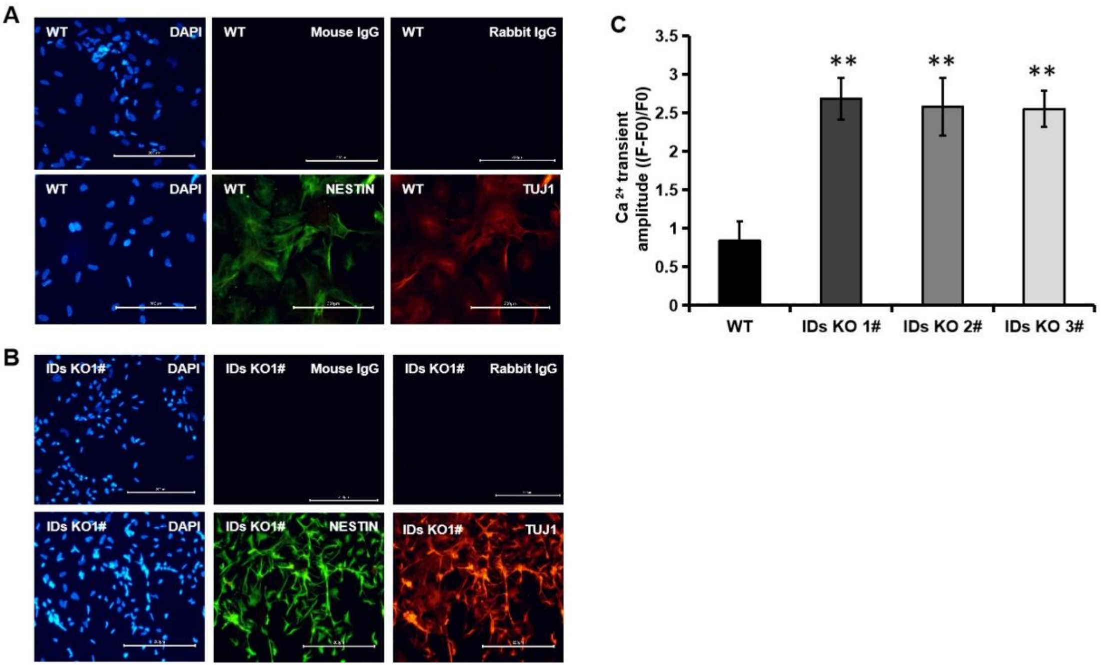

Fig. S3. Characterization with Neural markers and Ca^{2+} transient in differentiated WT and IDs KO cells.

A, B Immunofluorescence of NESTIN, TUJ1 and isotype IgG in neuron derived from WT and IDs KO hESCs, Bar=200 μm. **C** Quantitative analyses of Ca^{2+} transient amplitude in WT and IDs KO derived neuron (N=3), **p<0.01 compared with WT, data are presented as means ± SEM, one-way ANOVA followed by t-test.

Supplemental Figure 4

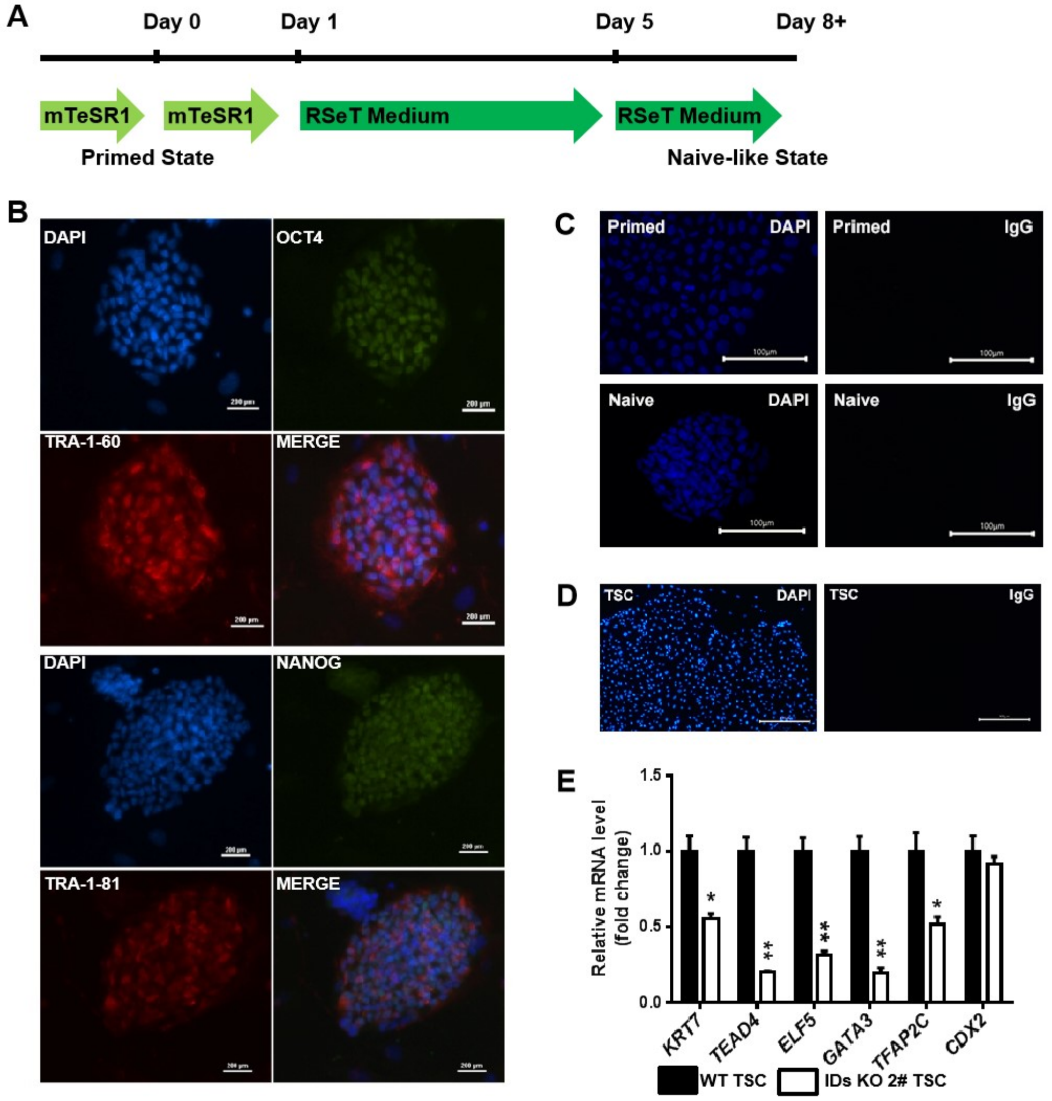

Fig. S4. Reset primed hESC to a naive-like state.

A Flow chart of the transition process from primed to naive state by RSeT medium. **B** Immunofluorescence staining of pluripotent markers in reset naive-like hESCs, bar=200μm. **C** Immunofluorescence of isotype IgG in primed and naive hESCs, bar=100μm. **D** Immunofluorescence of isotype IgG in TSC, bar=200μm. **E** RT-qPCR result of TSC-markers expression of WT and IDs KO 2# (N=3), compared with WT, *$p<0.05$; **$p<0.01$, data are presented as means ± SEM, t test.

Supplemental Figure 5

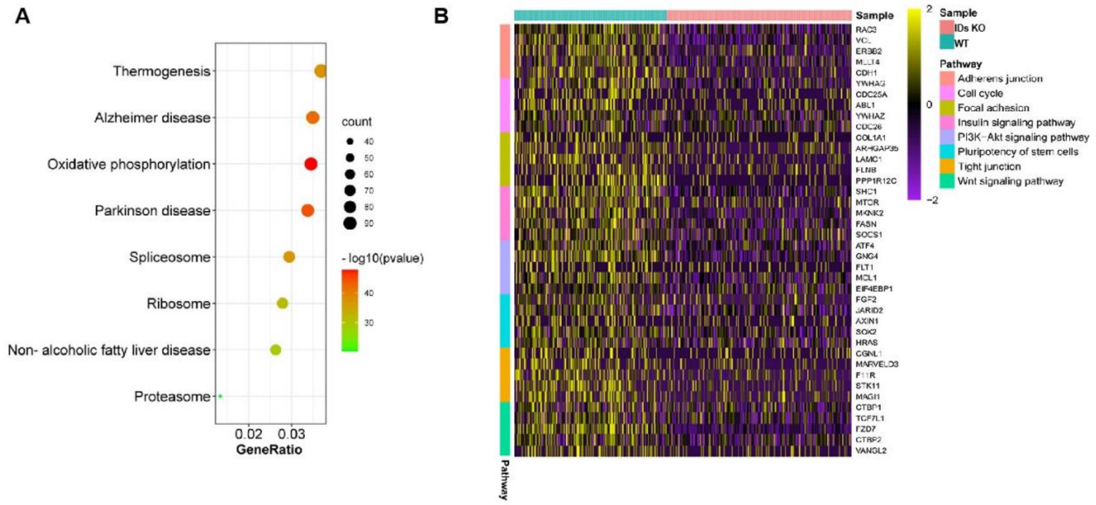

Fig. S5. KEGG analysis of Single cell RNA-seq of WT and IDs KO cells.

A KEGG analysis on up-regulation genes that IDs KO versus WT.

B Top 5 genes of down-regulated genes in IDs KO cells that enriched in the signal pathways by KEGG analysis.

Supplemental Figure 6

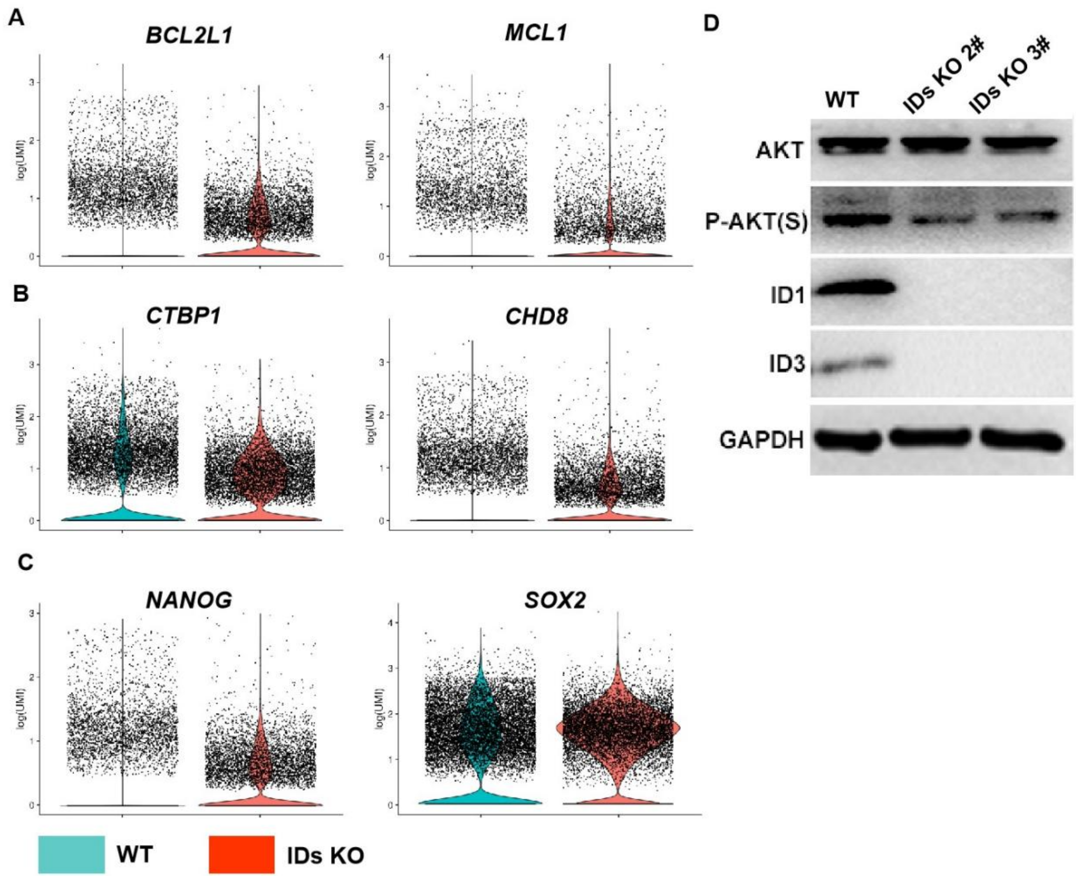

Fig. S6. The AKT pathway related gene expression of WT and IDs KO in naive-like state.

A PI3K/AKT signaling associated genes were shown in violin plots. **B** WNT signaling associated genes were shown in violin plots. **C** Pluripotent marker genes genes were shown in violin plots. **D** The AKT phosphorylation was detected by western blot of other IDs KO lines.

Supplemental Figure 7

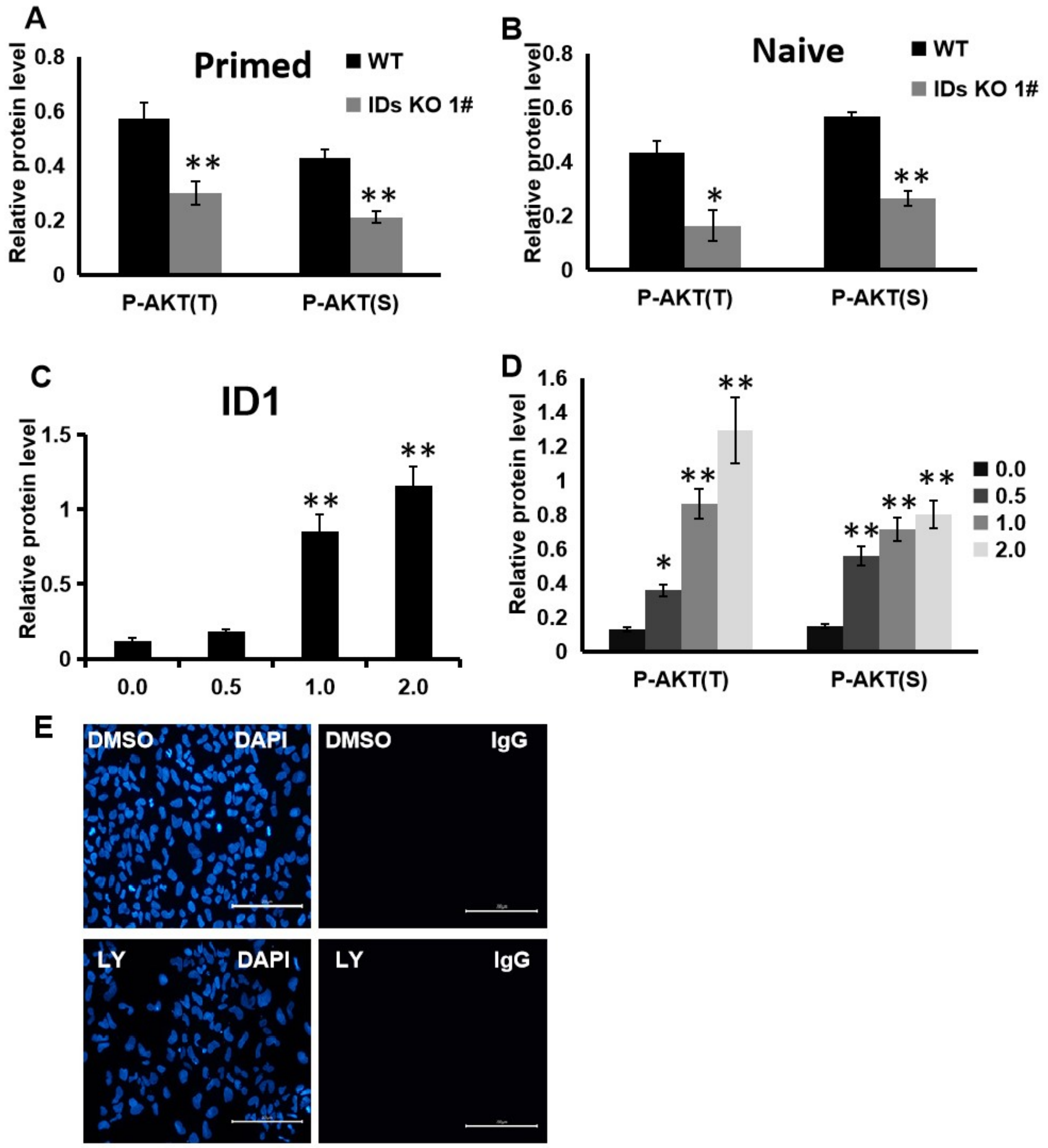

Fig. S7. Quantitative analyses of western blotting in figure 4 and immunofluorescence control in figure 5.

A, B, C were normalized to GAPDH, **D** were normalized to AKT in figure 4, (N=3), *p<0.05, **p<0.01 compared with WT, data are presented as means ± SEM, one-way ANOVA followed by t-test. **E** Immunofluorescence of isotype IgG in figure 5, bar=200μm.

Supplemental Figure 8

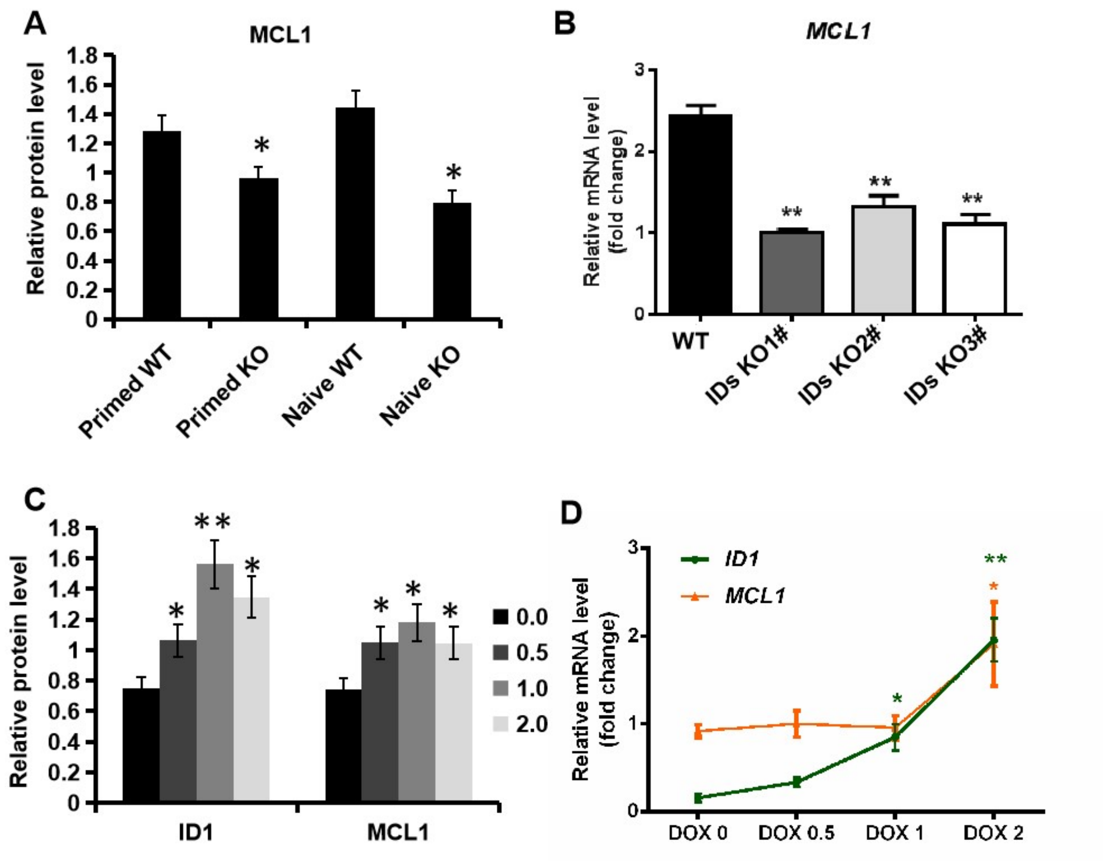

Fig. S8. The presence of ID facilitates the expression of MCL1.

A The mRNA level of *MCL1* of WT and IDs KO cells was determined by RT-PCR(N=3). **B** The mRNA level of *MCL1* when ID1 was induced by different concentration of DOX after 48h, N=3, compared with DOX 0. **C D** Quantitative analyses of western blotting in figure 6 **G** and **H** (N=3), normalized to GAPDH. *p<0.05; **p<0.01, data are presented as means ± SEM, one-way ANOVA followed by t test.

Supplemental Figure 9

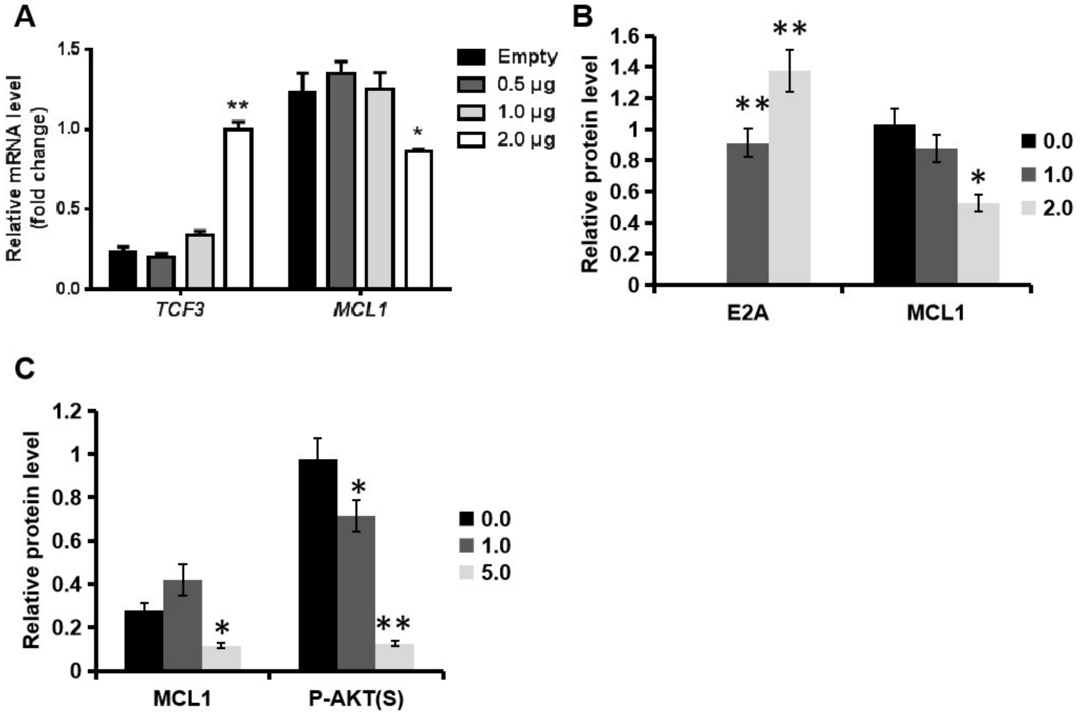

Fig. S9. Quantitative analyses of western blotting in figure 6.

A The mRNA level of TCF3 and MCL1 were detected by RT-qPCR after hESCs was transfected with empty and TCF3 over-expression plasmid (N=3), compared with WT. **B C** Quantitative analyses of western blotting in figure 6 **I** and **J** (N=3), **B** normalized to GAPDH, **C** MCL1 were normalized to GAPDH and P-AKT(S) were normalized to AKT, (N=3). *p<0.05, **p<0.01 compared with WT, data are presented as means ± SEM, one-way ANOVA followed by t-test.

Supplemental Figure 10

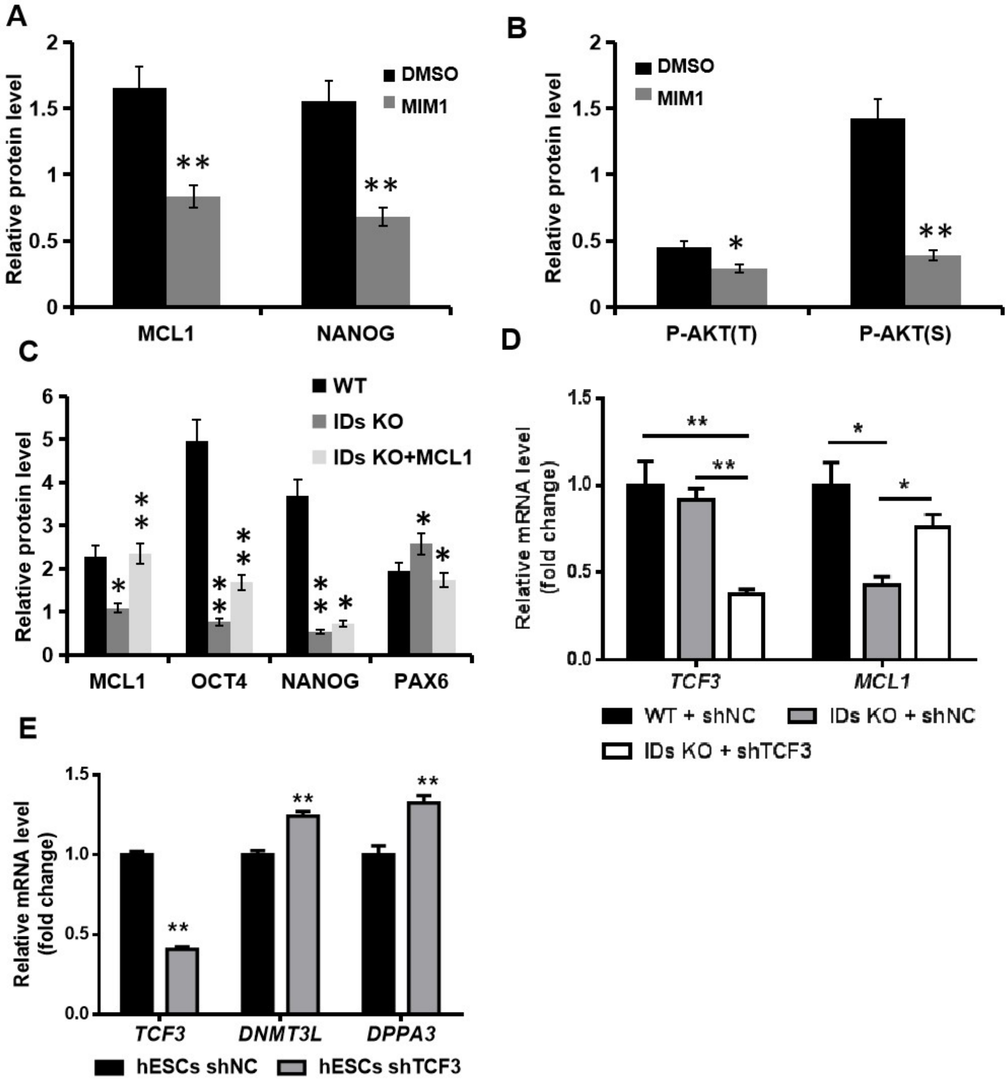

Fig. S10. Quantitative analyses of western blotting in figure 7.

A, normalized to GAPDH, **B** normalized to AKT, **C**, normalized to GAPDH, **D** Quantitative analysis of gene expression in WT and IDs KO naive-like hESC after transfected with shNC and shTCF3 (N=3), compared to WT+shNC, dates are presented as means ± SEM, *p<0.05, **p<0.01, one-way ANOVA followed by t-test. **E** RT-qPCR analyses of TCF3, DNMT3L, DPPA3 expression in hESCs after transfecting with shRNA (N=3), *p<0.05, **p<0.01 compared with shNC, data are presented as means ± SEM, one-way ANOVA followed by t-test.

Supplemental Tables

Table S1: List of primers used in this paper for qPCR.

Gene	Sequence(5’-3’)
qPCR-*OCT4*	sense：CTTCGCAAGCCCTCATTTCACCA<br>antisense：GCACTAGCCCCACTCCAACCTG
qPCR-*SOX2*	sense：AACCCCAAGATGCACAACTC<br>antisense：CGGGGCCGGTATTTATAATC
qPCR-*NANOG*	sense：TTCTGCTGAGATGCCTCACACGG<br>antisense：TCTTGACCGGGACCTTGTCTTCC
qPCR-*TERF1*	sense：AACAGCGCAGAGGCTATTATTC<br>antisense：CCAAGGGTGTAATTCGTTCATCA
qPCR-*PRMD14*	sense：TTCGTTCTGTACGGGGTCACT<br>antisense：TCTGCATGAGGCATAGACCTT
qPCR-*KLF2*	sense：CACCAAGAGTTCGCATCTGAAGG<br>antisense：TACATGTGCCGTTTCATGTGCAG
qPCR-*KLF17*	sense：GGGATGGTGCGATAGATTCA<br>antisense：GCCTCACCCTCACCTAACAA
qPCR-*DPPA3*	sense：ATCGGAAGCTTTACTCCGTCGAG<br>antisense：CCCTTAGGCTCCTTGTTTGTTGG
qPCR-*DPPA5*	sense：ACATCGAGCAGGTGAGCAAGG<br>antisense：CATGGCTTCGGCAAGTTTGAG
qPCR-*DNMT3L*	sense：CTGCGGAAGTCTCCAGGTTCA<br>antisense：GTAGCATCGGGTGCAATCAGG
qPCR-*TFCP2L1*	sense：CAGCCCGAGCACTACAACC<br>antisense：CTCCCAGCTTCCGATTCTCC
qPCR-*PAX6*	sense：ATGTGTGAGTAAAATTCTGGGCA<br>antisense：GCTTACAACTTCTGGAGTCGCTA
qPCR-*SOX1*	sense：AATTTTATTTTCGGCGTTGC<br>antisense：TGGGCTCTGTCTCTTAAATTTGT
qPCR-*DLK1*	sense：AGGGTCCCCTTTGTGACCA<br>antisense：GCAGGCCCGAACATCTCTATC

qPCR-*EOMES*	sense：CCGCCACCAAACTGAGATGA<br>antisense：ACATTTTGTTGCCCTGCATGT
qPCR-*T*	sense：TATGAGCCTCGAATCCACATAGT<br>antisense：CCTCGTTCTGATAAGCAGTCAC
qPCR-*SOX17*	sense：CGCACGGAATTTGAACAGTA<br>antisense：GGATCAGGGACCTGTCACAC
qPCR-*GATA4*	sense：CTCCATCCACCCTGTCCTC<br>antisense：ATTACGCAGTGATTATGTCCC
qPCR-*GAPDH*	sense：TCCAAAATCAAGTGGGGCGAT<br>antisense：TTCTAGACGGCAGGTCAGGTC
qPCR-*CDX2*	sense：TTCACTACAGTCGCTACATCACC<br>antisense：TTGATTTTCCTCTCCTTTGCTC
qPCR-*TEAD4*	sense：CAGGTGGTGGAGAAAGTTGAGA<br>antisense：GTGCTTGAGCTTGTGGATGAAG
qPCR-*TFAP2C*	sense：TCTTGGAGGACGAAATGAGATGG<br>antisense：GGGCTTCTTTGATGTAGTTCTGC
qPCR-*ELF5*	sense：AGTCTGCACTGACATTTTCTCATC<br>antisense：CAGAAGTCCTAGGGGCAGTC
qPCR-*KRT7*	sense：AGGATGTGGATGCTGCCTAC<br>antisense：CACCACAGATGTGTCGGAGA
qPCR-*GATA3*	sense：TGCAGGAGCAGTATCATGAAGCCT<br>antisense：GCATCAAACAACTGTGGCCAGTGA
qPCR-*CGB*	sense：ACCCTGGCTGTGGAGAAGG<br>antisense：ATGGACTCGAAGCGCACA
qPCR-*TCF3*	sense：TGGGCAGCAGTGGAAGCAG<br>antisense：GGGCTGAGGAGAAGGAGGATG
qPCR-*MCL1*	sense：GCCATCCCTGAACTCTTT<br>antisense：GGACTTCCTCCCACCTCT
qPCR-*ID1*	sense：GGCTGTTACTCACGCCTCAAG<br>antisense：CCAACTGAAGGTCCCTGATGTAG
qPCR-*ID3*	sense：CTGGACGACATGAACCACTG<br>antisense：GTAGTCGATGACGCGCTGTA

Table S2: List of antibodies used in this paper for immunofluorescence and western blot

Name	Company	Ref number
Anti-OCT4	Abcam	ab18976
NANOG Antibody	CST	3580S
TRA-1-60 mouse mAb	CST	4746S
TRA-1-81 mouse mAb	CST	4745S
Rabbit Monoclonal Anti- Human ID1	BIOCHECK	BCH-1/195-14
Rabbit Monoclonal Anti- Human ID3	BIOCHECK	BCH-4/17-3
AKT (pan) (C67E7) Rabbit mAb	CST	4691S
Phospho-AKT (Thr308) Rabbit mAb	CST	13038S
Phospho-AKT (Ser473) Rabbit mAb	CST	4060S
E2A (D2B1) Rabbit mAb	CST	12258S
Rabbit Control IgG	Abclonal	AC005
MCL1 Rabbit mAb	Abclonal	A10993
Anti-GAPDH Mouse mAb	TransGen	HC301-01
ELF5 Rabbit pAb	Abclonal	A7181
KRT7 Rabbit pAb	Abclonal	A2574
p63 Rabbit pAb	Abclonal	A12937
PAX6 Rabbit pAb	Abclonal	A7334
NESTIN Mouse mAb	CST	33475S
βIII-TUBULIN Rabbit mAb	Abclonal	A17913
Alexa Fluor 488 donkey anti-mouse	Invitrogen	A21202

Alexa Fluor 488 donkey anti-rabbit	Invitrogen	A21206
Alexa Fluor 647 donkey anti-mouse	Invitrogen	A31571
Alexa Fluor 647 donkey anti-rabbit	Invitrogen	A31573
HRP Goat Anti-Rabbit IgG	Abclonal	AS014
HRP Goat Anti-Mouse IgG	Abclonal	AS003
