# Supplementary material for: ID proteins promote the survival and primed-to-naive transition of human embryonic stem cells through TCF3-mediated transcription
Source: Cell Death Dis. 2022 Jun 15;13(6):549. doi: 10.1038/s41419-022-04958-8 (PMC9198052; doi:10.1038/s41419-022-04958-8)

ORIGINAL IMAGES OF WESTERN BLOT

For Figure S1B

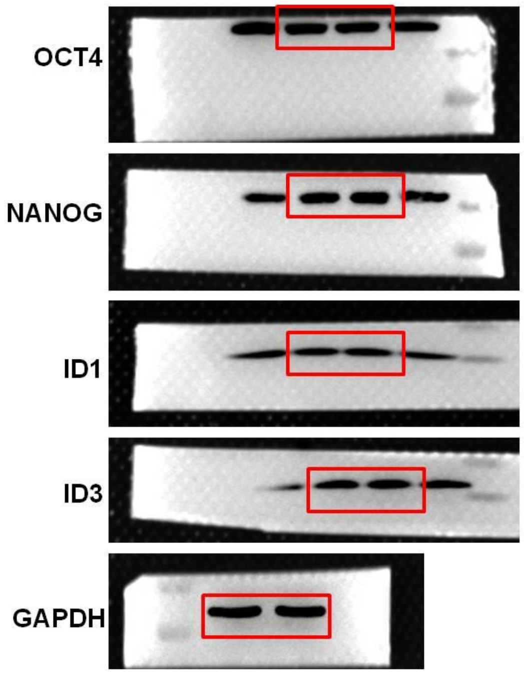

For Figure S1C

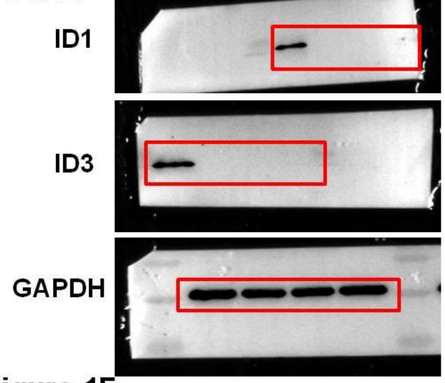

For Figure 1F

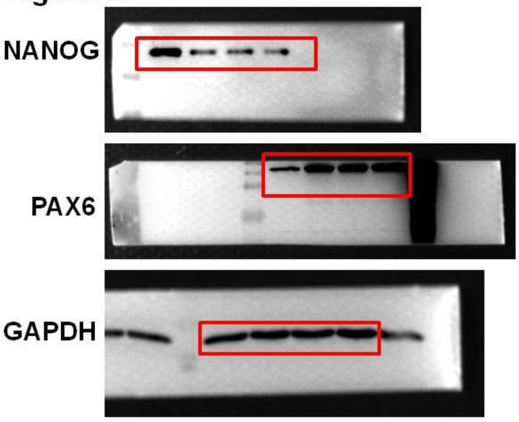

For Figure 4G

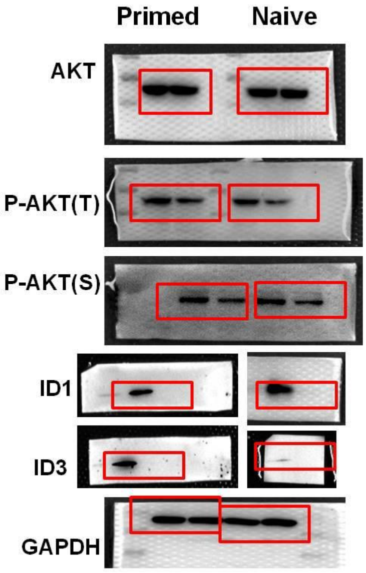

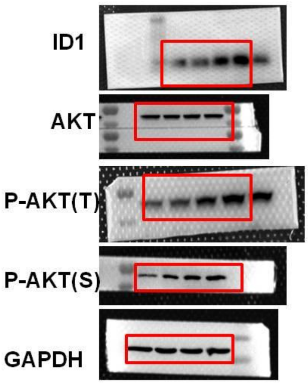

For Figure S6D

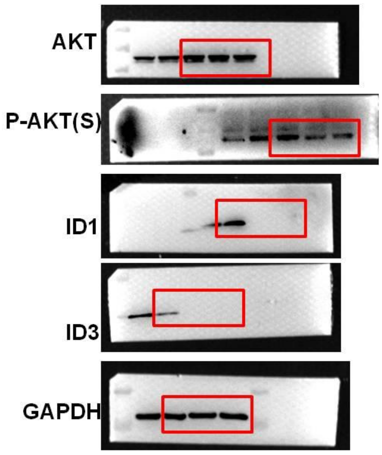

For Figure 6A

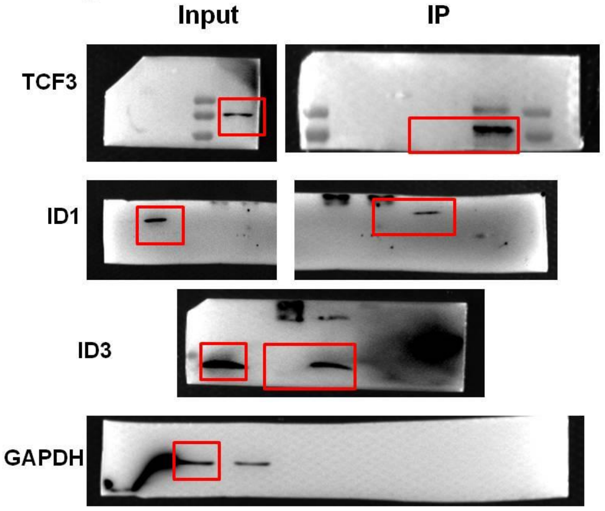

For Figure 6G

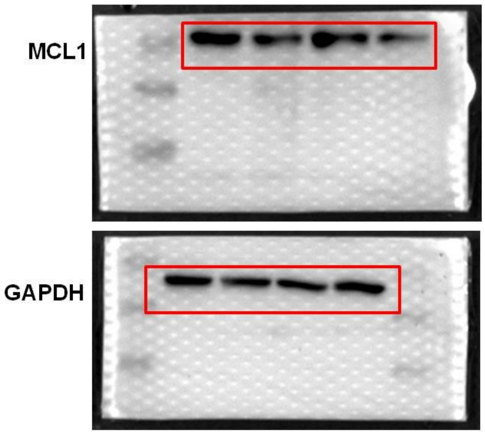

For Figure 6H

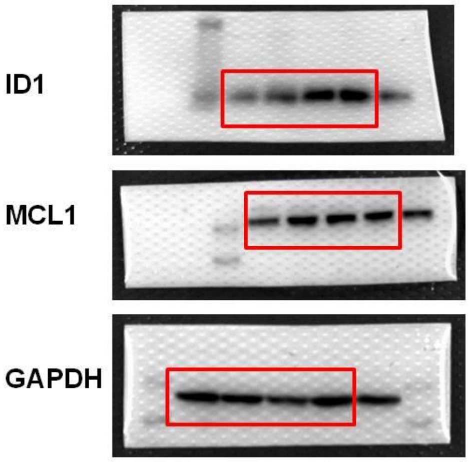

For Figure 6I

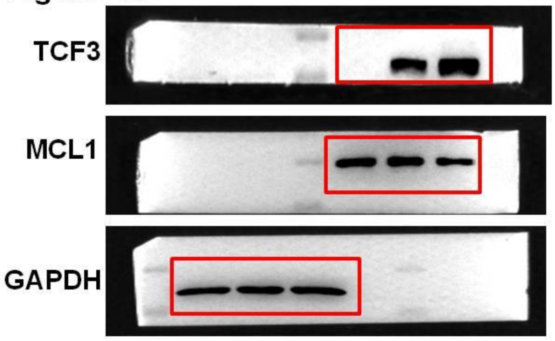

For Figure 6J

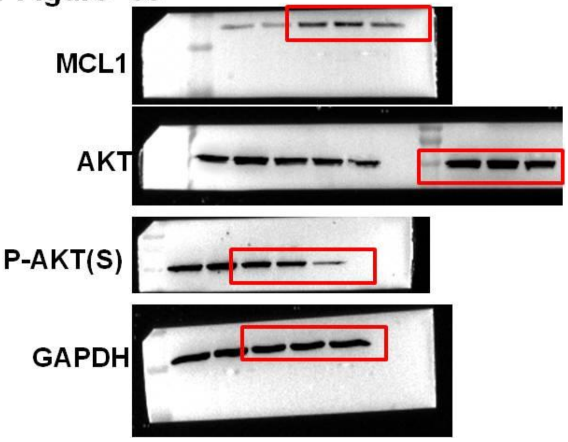

For Figure 7E

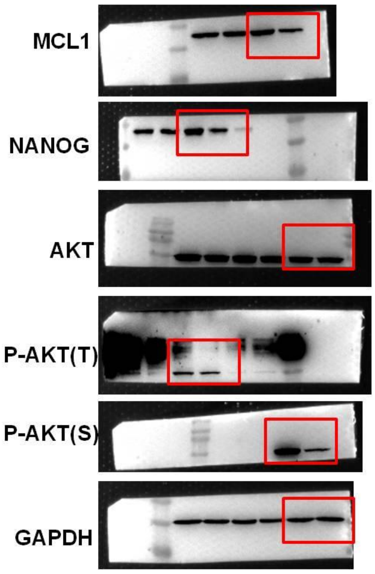

For Figure 7H

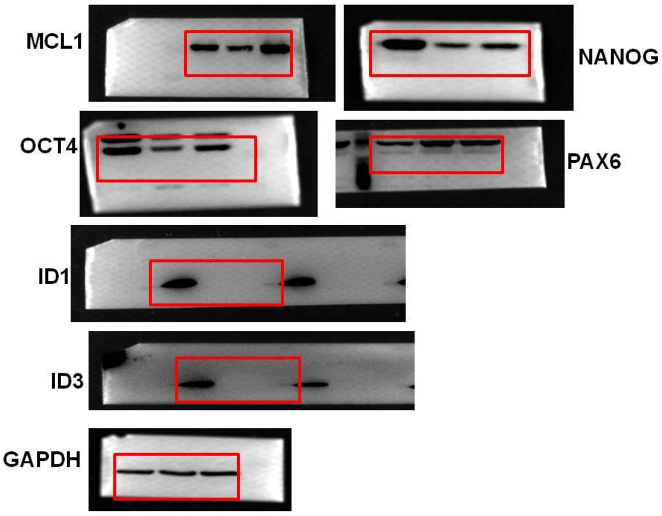

Supplement: Supplementary file 4 — Original Data File [file 41419_2022_4958_MOESM4_ESM.pdf]
